# Computed Tomography elucidates ontogeny within the basal therapsid clade Biarmosuchia

**DOI:** 10.7717/peerj.11866

**Published:** 2021-08-26

**Authors:** Aliénor Duhamel, Julien Benoit, Michael Day, Bruce Rubidge, Vincent Fernandez

**Affiliations:** 1 ENS de Lyon, CNRS, UMR 5276, LGL-TPE, Université Claude Bernard (Lyon I), Lyon, France; 2 Evolutionary Studies Institute, University of the Witwatersrand, Johannesburg, South Africa; 3 School of Geosciences, University of the Witwatersrand, Johannesburg, South Africa; 4 Department of Earth Sciences, Natural History Museum, London, United Kingdom; 5 European Synchrotron Radiation Facility, Grenoble, France

**Keywords:** Synapsida, Therapsida, Biarmosuchia, Ontogeny, CT-scan

## Abstract

Biarmosuchia is a clade of basal therapsids that includes forms possessing plesiomorphic ‘pelycosaurian’ cranial characters as well as the highly derived Burnetiamorpha which are characterised by cranial pachyostosis and a variety of cranial bosses. Potential ontogenetic variation in these structures has been suggested based on growth series of other therapsids with pachyostosed crania, which complicates burnetiamorph taxonomic distinction and thus it is essential to better understand cranial ontogeny of the Burnetiamorpha. Here, three new juvenile biarmosuchian skulls from the late Permian of South Africa are described using X-ray micro computed tomography (CT). We found that juvenile biarmosuchians are distinguished from adults by their relatively large orbits, open cranial sutures, and incomplete ossification of the braincase and bony labyrinth. Also, they manifest multiple centres of ossification within the parietal and preparietal bones. CT examination reveals that the holotype of *Lemurosaurus pricei* (BP/1/816), previously alleged to be a juvenile, shows no evidence of juvenility and is thus probably an adult. This suggests that the larger skull NMQR 1702, previously considered to be an adult *L. pricei*, may represent a new taxon. This study provides, for the first time, a list of characters by which to recognise juvenile biarmosuchians.

## Introduction

Largely because of the small numbers of complete specimens, the Biarmosuchia is a poorly-understood group of mid-late Permian therapsids in terms of phylogeny, ontogeny, locomotion (paucity of post-crania) and paleobiology (Hopson & Barghusen, 1986; Rubidge & Sidor, 2001; Kemp, 2006). Broom (1913) was the first to describe a member of this clade, *Ictidorhinus martinsi*, from South Africa. Over the past two decades several new biarmosuchian specimens have been described and historical specimens have been re-assessed (Rubidge & Kitching, 2003; Sidor, 2003; Rubidge, Sidor & Modesto, 2006; Sidor & Rubidge, 2006; Kruger et al., 2015; Day, Rubidge & Abdala, 2016; Kammerer, 2016; Benoit et al., 2017b; Day et al., 2018; Kulik & Sidor, 2019), but the systematics of the group remains uncertain as all described genera are monospecific and most species are represented by only one specimen (cranial material). Even though the Biarmosuchia is considered a monophyletic group (Rubidge & Sidor, 2001; Liu, Rubidge & Li, 2009; Day et al., 2018), taxonomic diversity and phylogenetic positions of genera are not yet well understood as some published phylogenetic analyses are still contradictory (Day, Rubidge & Abdala, 2016; Kammerer, 2016; Day et al., 2018).

Thirty-one biarmosuchian genera have been described, mainly based on cranial material, from different regions of Pangea (Malawi, Zambia, Tanzania, Zimbabwe, and Russia). However, some of these are not considered valid or diagnosable. Twelve valid genera are from the main Karoo Basin of South Africa (Rubidge & Kitching, 2003; Sidor, 2003; Sidor & Welman, 2003; Sidor, Hopson & Keyser, 2004; Rubidge, Sidor & Modesto, 2006; Smith, Rubidge & Sidor, 2006; Sidor & Smith, 2007; Kruger et al., 2015; Kammerer, 2016; Day et al., 2018) where they have been recovered from all Permian tetrapod-defined biozones of the Beaufort Group except the *Eodicynodon* Assemblage Zone (AZ) (Day, Rubidge & Abdala, 2016).

One of the best-represented biarmosuchian subclades, both in numbers of specimens and quality of preservation, is the Burnetiamorpha. This group includes *Lemurosaurus pricei* as the basal-most taxon (Sidor & Welman, 2003; Sidor & Smith, 2007; Kruger et al., 2015; Kammerer, 2016; Kammerer & Sidor, 2021) and the Burnetiidae is the most derived subclade (Rubidge & Kitching, 2003; Sidor, 2003; Sidor & Welman, 2003; Sidor, Hopson & Keyser, 2004; Rubidge, Sidor & Modesto, 2006; Smith, Rubidge & Sidor, 2006; Sidor & Smith, 2007; Kruger et al., 2015; Kammerer, 2016; Day et al., 2018; Kammerer & Sidor, 2021). Most burnetiamorph genera are represented by a single specimen, which limits understanding of ontogenetic development and sexual dimorphism (Sigogneau, 1970; Sidor & Welman, 2003; Kruger et al., 2015; Kulik & Sidor, 2019).

Previous research on ontogenetic series of some non-biarmosuchian therapsid taxa provided insight on their palaeobiology. Allometric studies have been undertaken on various therapsid groups to evaluate and study ontogenetic patterns (Huttenlocker & Abdala, 2015; Jasinoski, Abdala & Fernandez, 2015; Jasinoski & Abdala, 2017a; Krone, Kammerer & Angielczyk, 2019). Using allometric and multivariate analyses Jasinoski & Abdala (2017a) identified ontogenetic modifications and sexual dimorphism in the cynodont *Galesaurus planiceps* and found that a few craniomandibular features, including the shape of the sutures, change during ontogeny. Moreover, the discovery of juvenile specimens of *Thrinaxodon liorhinus, Galesaurus planiceps*, and *Kayentatherium wellesi* in association with adult individuals enabled research on anatomical features linked to ontogeny and led to the conclusion that these species provided parental care (Jasinoski & Abdala, 2017b; Hoffman & Rowe, 2018, but see Benoit, 2019). Ontogenetic studies on South African and Russian dinocephalians have also revealed allometric growth in the length of the skull compared to the diameter of the orbit, and some specimens like *Stenocybus* and *Sinophoneus* are likely to be part of the same ontogenetic series (Ivakhnenko, 2003, 2008; Kammerer, 2011; Kruger, Rubidge & Abdala, 2017). It has also been demonstrated that cranial ornamentation developed during life in *Biarmosuchus* and several dinocephalians such as *Estemmenosuchus* (Ivakhnenko, 2008). Understanding burnetiamorph cranial ontogenesis would thus possibly provide a path to understand their elusive palaeobiology.

To investigate biarmosuchian ontogeny we studied two new, apparently juvenile, biarmosuchian specimens (CGS MJF 22 and SAM-PK-K11126), and three already described putative juvenile specimens: the holotype of *Rubidgina angusticeps* (RC 55), *Lende chiweta* (MAL290), the holotype of *Lemurosaurus pricei* (BP/1/816), and a specimen (NMQR 1702) previously considered to represent a more mature specimen of *L*. *pricei* (Sidor & Welman, 2003). Micro CT was undertaken to determine histological and anatomical clues to characterise ontogenetic stages in this clade. Our results support the non-juvenile status of BP/1/816 and the taxonomic status of *Lemurosaurus pricei* is revised accordingly.

## Materials and Methods

The specimens described in this paper were chosen either because they have been described as juveniles, or because they have not yet been described and exhibit characters suggesting that they are juveniles.

### New material

CGS MJF 22 is an almost complete skull, collected in 1980 on the farm Amsterdam, in the Victoria West district of the Northern Cape, in strata that appear on the geological map (Geological Survey, 1989) as belonging to the Poortjie Member (presumably the *Lycosuchus*-*Eunotosaurus* Subzone of the *Endothiodon* AZ) of the Teekloof Formation (Day & Smith, 2020). The biostratigraphic provenance of this specimen remains uncertain as associated material is fragmentary and uninformative, and the lithostratigraphic units as mapped in the Victoria West area are not necessarily consistent with the biostratigraphic relationships established in their type areas (Day & Rubidge, 2019).

Specimen SAM-PK-K11126 includes a snout with an almost complete palate, a fragment of the pineal region, a fragment of the occiput around the foramen magnum, and fragments of the lower jaw. Eleven vertebrae (dorsal and sacral) and the pelvis are preserved in articulation. The proximal epiphysis of a femur, one indeterminate long bone diaphysis, and three indeterminate bony fragments are also preserved. Only the cranial material is described here for comparison; the postcranium will be the subject of a future study. Specimen SAM-PK-K11126 was collected in 2012 by Zaituna Skosan on the farm Reiersvlei, in the Beaufort West district of the Western Cape. It was found in the upper part of the Poortjie Member (*Endothiodon* AZ) *sensu* (Day & Smith, 2020).

### Previously-described material

Specimen RC 55 comprises an almost complete skull. It was collected in 1940 by a party led by Croonie Kitching on the farm Wellwood in the Graaff-Reinet district of the Eastern Cape, from rocks of the Balfour Formation, likely *Cistecephalus* AZ, ~256 Ma (Day et al., 2015). It was described by Broom (1942) as the holotype of *Rubidgina angusticeps*, initially considered a gorgonopsian, and later assigned to the “Ictidorhinidae” by Sigogneau (1970). Most recently, RC 55 was identified as a potential juvenile of the non-burnetiamorph biarmosuchian *Herpetoskylax hopsoni* (Sidor & Rubidge, 2006).

BP/1/816 is a relatively complete skull and lower jaw, collected in 1948 by James Kitching on the farm Dorsfontein in the Graaff-Reinet district in the lower Balfour Formation (*Cistecephalus* AZ). It was described by Broom (1949), who designated it as the holotype of *Lemurosaurus pricei* and was later part of Sigogneau’s (1970) PhD dissertation. *Lemurosaurus pricei* was also placed among Ictidorhinidae by Sigogneau (1970) but was considered as a possible juvenile burnetiamorph by Sidor & Welman (2003).

Specimen NMQR 1702 is a well-preserved skull and lower jaw collected in 1974 by a team from the National Museum, Bloemfontein, on the farm Petersburg in the Graaff-Reinet district. Although the precise locality of the find is not known and the farm Petersburg straddles the escarpment and a significant stratigraphic thickness, the specimen most likely comes from the lower Balfour Formation (*Cistecephalus* AZ) (Sidor & Welman, 2003). The specimen was described and referred to *L. pricei* by Sidor & Welman (2003).

Specimen MAL290 (holotype of *Lende chiweta*) is a well-preserved skull and lower jaw collected in the Chiweta Beds of Malawi dated from the same period as the South African *Cistecephalus* AZ. The specimen was described by Kruger et al. (2015) and classified as a derived burnetiamorph.

### Preparation

Specimen CGS MJF 22 was prepared mechanically at the Evolutionary Studies Institute by Charlton Dube using a compressed air driven air-scribe equipped with specially adapted and sharpened tungsten carbide tips. Paraloid diluted with acetone was used as an adhesive. For SAM-PK-K11126, partial preparation was previously undertaken at Iziko Museum in Cape Town. Specimen RC 55 had been previously prepared using a vibro-tool fitted with gramophone needles.

### Scanning

To enhance understanding of internal cranial morphology and sutural patterns, CGS MJF22 and BP/1/816 (holotype of *L. pricei*) were scanned at the ESI using X-ray microCT with a Nikon Metrology XTH 225/320 LC (scanning parameters: 0.0445 mm, 185 kV, 185 μA and 0.050 mm, 110 kV, 150 μA respectively). Specimen SAM-PK- K11126 was scanned during two sessions at the same CT facility. The skull roof, occiput, and lower jaw were scanned during the first session and the snout during the second one (scanning parameters: 0.034 mm, 155 kV, 235 μA, and 0.03448 mm, 155 kV, 235 μA for each respective session). Specimens RC 55 and MAL 290 were also scanned at the ESI using the following scanning parameters: 0.0545 mm, 70 kV and 160 μA; and 0.08 mm, 105kV and 160 μA respectively. Specimen NMQR 1702 was scanned at the ID17 beamline of the European Synchrotron Radiation Facility (ESRF, Grenoble, France), using propagation phase contrast synchrotron X-ray micro computed tomography. The beamline setup consisted of a monochromatic beam of 130 keV (Si 111 double bent Lauë monochromator); filtration with 4 mm of aluminium and 1 mm of copper; a sample-detector distance of 10.9 m; an indirect detector (2 mm thick LuAG scintillator, 0.26× optical magnification and a FReLoN-2k camera), recording imaging with an isotropic pixel size of 0.054 mm. The data acquisition was done following the so-called attenuation protocol (Carlson et al., 2011), placing the specimen in a plastic tube filled with aluminium balls 5 mm in diameter, and consisted of 4,998 projections recorded over a rotation of 360°, an exposure time of 0.6 s each. Additionally, the centre of rotation was shifted to increase the reconstructed horizontal field of view. Given the limited vertical size of the X-ray beam (~8 mm), 71 scans were necessary to cover the full length of the skull, moving the specimen by 5 mm between each acquisition. The CT reconstructions were done with the software PyHST2, using the single-distance phase retrieval approach (Paganin et al., 2002; Mirone et al., 2014). The resulting 32-bit data were converted to a stack of 16 bits tiff using 0.02% saturation values from the 32-bit 3D histogram generated by PyHST2. Raw CT data are stored at the Evolutionary Studies Institute and are available upon request to the authors by email: alienor.duhamel@ens-lyon.org.

Three-dimensional reconstructions and visualization of CT data of all specimens were generated using AVIZO 9.0 (FEI VSG, Hillsboro, OR, USA). Three-dimensional renderings were obtained using manual segmentation with AVIZO 9.0 (FEI VSG, Hillsboro, OR, USA). Surface files from the 3D reconstruction are available in the Supplemental Information and on the website MorphoBank under the project number P4003 (https://morphobank.org/index.php/Projects/ProjectOverview/project_id/4003).

## Description

This section presents a full updated anatomical description of three of the five above mentioned skulls. Specimens NMQR 1702, BP/1/816 and MAL290 are not fully redescribed here as (i) sufficiently detailed descriptions of their external cranial anatomy are already available in the published literature (Sigogneau, 1970; Sidor & Welman, 2003; Kruger et al., 2015; Benoit et al., 2017b) and (ii) the CT images did not enable isolation of all the bones using digital segmentation and therefore do not significantly add to previous descriptions. Only new observations enabled by CT scanning are discussed in this paper.

BIARMOSUCHIA Sigogneau-Russell, 1989

BIARMOSUCHIA gen. et sp. indet.

**Material**–RC 55, the holotype of *Rubidgina*, comprises a nearly complete laterally compressed skull. The specimen was considered a possible juvenile of *Herpetoskylax hopsoni* by Sidor & Rubidge (2006). RC 55 can be identified as a biarmosuchian by the presence of a squamosal with a long ventral ramus and an elongated zygomatic process under the orbit, but we consider it indeterminate within this clade because of its juvenile condition and lack of diagnostic characters.

**Figure 1:**
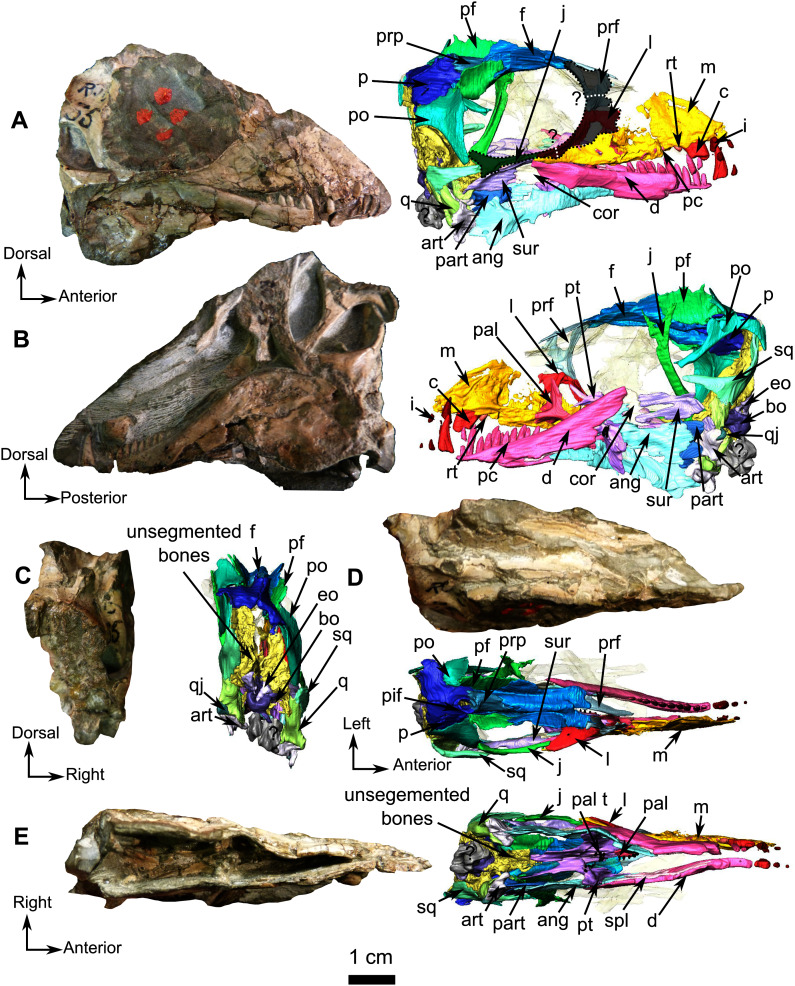
Holotype of *Rubidgina angusticeps*, here considered Biarmosuchia indet., RC 55. Photograph (left) and 3D rendering (right). (A) Right lateral view. (B) Left lateral view. (C) Occipital view. (D) Dorsal view. (E) Ventral view. Anatomical Abbreviations –ang, angular; art, articular; aso, anterior extension of the supraoccipital; bo, basioccipital; c, caniniform tooth; co, occipital condyle; cor, coronoid; d, dentary; ect, ectopterygoid; eo, exoccipital; f, frontal; fe, temporal fenestra; fm, foramen magnum; i, incisiform tooth; j, jugal; l, lacrimal; m, maxilla; n, nasal; o, orbit; op, opisthotic; p, parietal; pa, anterior portion of the parietal; pac, caudal portion of the parietal; pal, palatine; pal t, palate teeth; pao, paroccipital process; part, pre-articular; pbs, parabasisphenoid; pc, postcanine; pf, postfrontal; pif, pineal foramen; pl, lateral portion of the parietal; pm, premaxilla; po, postorbital; pp, postparietal; prf, prefrontal; pro, prootic; prp, preparietal; pt, pterygoids; ptf, post-temporal fenestra; q, quadrate; qj, quadratojugal; rt, replacement tooth; sb, supraorbital boss; scl, sclerotic ring; smx, septomaxilla; so, supraoccipital; spl, splenial; st, stapes; sq, squamosal; sur surangular; t, tabular; v, vomer. Pictures by A. Duhamel. 3D reconstructions were made by A. Duhamel and belong to the University of the Witwatersrand. Scale bar = 1 cm.

**Description**–This specimen is an almost complete skull with lower jaws (Fig. 1). The orbit is relatively large (3 cm diameter) in comparison to the skull length (7.5 cm), and the skull roof is flat and does not show any sign of bosses, ridges, or pachyostosis (Duhamel et al., 2016; Duhamel, Benoit & Rubidge, 2018; Kulik & Sidor, 2019), consistent with a juvenile status or a basal phylogenetic position (Sidor & Rubidge, 2006). In lateral view, the skull has a triangular outline (Fig. 1). The specimen is poorly preserved, being laterally compressed with the left side eroded. The anterior snout has been eroded such that the premaxilla, septomaxilla, part of the maxilla, incisors, and a large part of the nasals are damaged (Fig. 1). As a result of diagenesis, most occipital bones are crushed, and the posterior margin of the right temporal fenestra is missing while the left is distorted (Fig. 1). Because sutures are difficult to recognise and many bones are concealed by matrix, the following description is mostly based on the 3D renderings. As a result of the poor visibility of the sutures, the postparietal, supraoccipital, tabular, opisthotic, prootic, parabasipshenoid and stapes could not be separated digitally but are definitely preserved on the specimen as seen on the “unsegmented bones” section (Fig. 1C). Both lower jaw rami are present and articulated to the skull (Fig. 1).

### Skull

**Figure 2:**
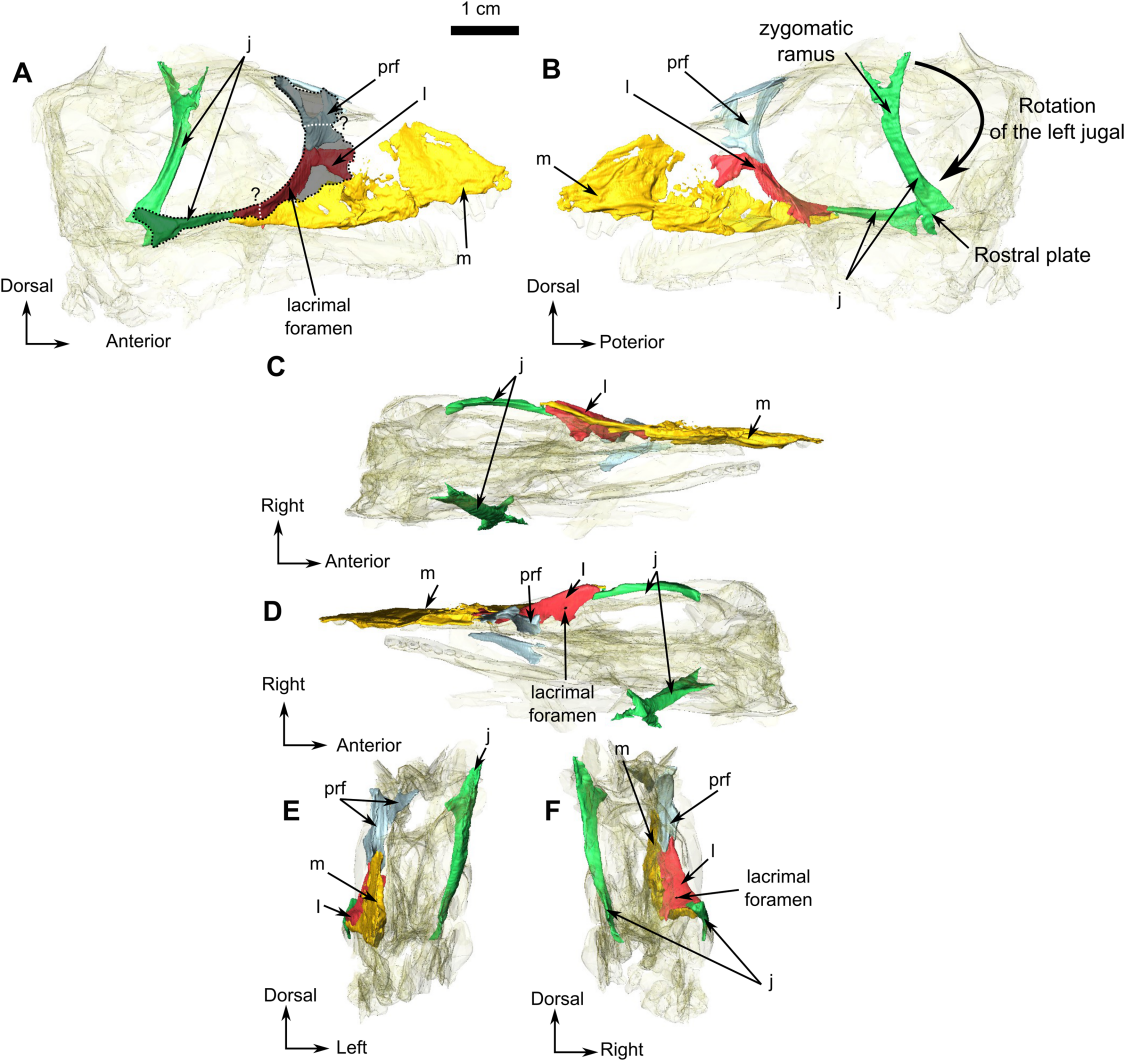
Holotype of *Rubidgina angusticeps*, here considered Biarmosuchia indet., RC 55. 3D rendering of the lateral portion of the skull. (A) Right lateral view. (B) Medial view. (C) Ventral view. (D) Dorsal view. (E) Anterior view. (F) Occipital view. Anatomical Abbreviations—ang, angular; art, articular; aso, anterior extension of the supraoccipital; bo, basioccipital; c, caniniform tooth; co, occipital condyle; cor, coronoid; d, dentary; ect, ectopterygoid; eo, exoccipital; f, frontal; fe, temporal fenestra; fm, foramen magnum; i, incisiform tooth; j, jugal; l, lacrimal; m, maxilla; n, nasal; o, orbit; op, opisthotic; p, parietal; pa, anterior portion of the parietal; pac, caudal portion of the parietal; pal, palatine; pal t, palate teeth; pao, paroccipital process; part, pre-articular; pbs, parabasisphenoid; pc, postcanine; pf, postfrontal; pif, pineal foramen; pl, lateral portion of the parietal; pm, premaxilla; po, postorbital; pp, postparietal; prf, prefrontal; pro, prootic; prp, preparietal; pt, pterygoids; ptf, post-temporal fenestra; q, quadrate; qj, quadratojugal; rt, replacement tooth; sb, supraorbital boss; scl, sclerotic ring; smx, septomaxilla; so, supraoccipital; spl, splenial; st, stapes; sq, squamosal; sur surangular; t, tabular; **v**, vomer. 3D reconstructions were made by A. Duhamel and belong to the University of the Witwatersrand. Scale bar = 1 cm.

The anterior portion of the skull is damaged, including the premaxilla and septomaxilla. The full extent of the maxilla is difficult to evaluate but despite its weathered surface it is clear that it is the largest bone of the lateral surface of the snout (Figs. 1 and 2). Posterodorsally, the maxilla has a long and oblique sutural contact with the lacrimal on the anterior orbital rim. Posteroventrally, the maxilla extends on the zygomatic arch as a long and thin process that reaches the anterior end of the jugal at the level of the dentary/surangular suture (Figs. 1A and 2). The medial surface of the maxilla is excavated by a triangular maxillary sinus (Fig. 2B). The facial area of RC 55 is crushed, and it is thus difficult to position the prefrontal, lacrimal and jugal sutures with accuracy. The following description is based on an interpretation that we estimate to be the most probable, however a possible second interpretation is shown in Figs. 1A and 2A. The lacrimal, as preserved, is a thin strip-like bone limited to the anteroventral margin of the orbit (Figs. 1 and 2) but, because the facial process is damaged, it is difficult to estimate the anterolateral extension of the bone. Dorsally the lacrimal has a short contact with the prefrontal (Figs. 2A and 2B). Posteroventrally, at the base of the zygomatic arch, the lacrimal meets the jugal and the maxilla in a tripartite suture (Fig. 2A). Medially, the lacrimal has a sutural contact with the palatine within the orbit (Fig. 1B). Two nasolacrimal foramina are present on the lacrimal: one medially positioned (Fig. 2F) and the second is on the lateral surface (Fig. 2A).

The prefrontal is a small bone forming the anterodorsal margin of the orbit (Figs. 2A and 2B). Although the right prefrontal has a flat anterodorsal extension (Figs. 2A and 2B), this unusual morphology could be the result of deformation or an artefact of segmentation. In dorsal view, the left prefrontal is triangular with posterolateral and posteromedial extensions (Fig. 2D). The anterior margin of the frontal is positioned between these two small extensions (Fig. 1D).

**Figure 3:**
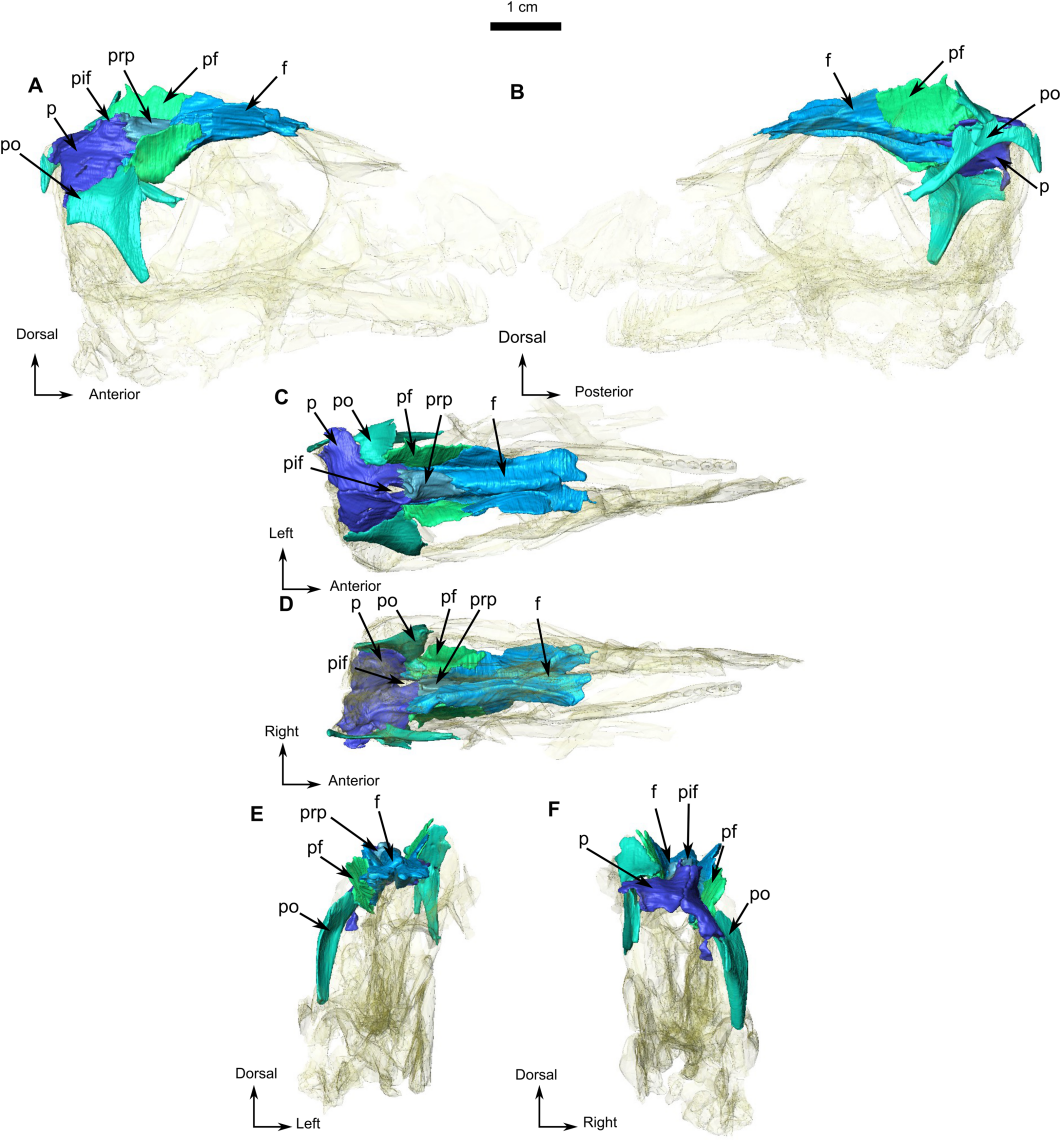
Holotype of *Rubidgina angusticeps*, here considered Biarmosuchia indet., RC 55. 3D rendering of the dorsal portion of the skull roof of RC 55. (A) Right lateral view. (B) Left lateral view. (C) Dorsal view. (D) Ventral view. (E) Anterior view. (F) Occipital view. Anatomical Abbreviations—ang, angular; art, articular; aso, anterior extension of the supraoccipital; bo, basioccipital; c, caniniform tooth; co, occipital condyle; cor, coronoid; d, dentary; ect, ectopterygoid; eo, exoccipital; f, frontal; fe, temporal fenestra; fm, foramen magnum; i, incisiform tooth; j, jugal; l, lacrimal; m, maxilla; n, nasal; o, orbit; op, opisthotic; p, parietal; pa, anterior portion of the parietal; pac, caudal portion of the parietal; pal, palatine; pal t, palate teeth; pao, paroccipital process; part, pre-articular; pbs, parabasisphenoid; pc, postcanine; pf, postfrontal; pif, pineal foramen; pl, lateral portion of the parietal; pm, premaxilla; po, postorbital; pp, postparietal; prf, prefrontal; pro, prootic; prp, preparietal; pt, pterygoids; ptf, post-temporal fenestra; q, quadrate; qj, quadratojugal; rt, replacement tooth; sb, supraorbital boss; scl, sclerotic ring; smx, septomaxilla; so, supraoccipital; spl, splenial; st, stapes; sq, squamosal; sur surangular; t, tabular; v, vomer. 3D reconstructions were made by A. Duhamel and belong to the University of the Witwatersrand. Scale bar = 1 cm.

The frontal is a long and thin bone forming the anterior half of the dorsal margin of the orbit (Fig. 1). It sutures with the prefrontal anteriorly, the preparietal posteromedially, the postfrontal posterolaterally, and the parietal posteriorly (Figs. 1 and 3C). The suture with the nasal is not preserved. In lateral view, a posterolateral process of the frontal extends lateral to the postfrontal on the orbital rim (Fig. 3B). Another thin process separates the postfrontal and the preparietal and meets the parietal posteriorly with a short contact in line with the anterior margin of the pineal foramen (Fig. 3C). As in most biarmosuchians, a longitudinal depression on the frontal separates the low orbital rim from a rounded midline ridge (Figs. 3E and 3F). Given the lateral compression of the specimen, the depth of this depression might have been exaggerated by post-mortem damage. In ventral view (Fig. 3D), the frontal bears a thick longitudinal ridge that curves medially and (on the left frontal) continues posteriorly onto the parietal where it disappears caudally (Fig. 3D).

The preparietal is a small unpaired and almost rectangular bone located on the dorsal-most point of the skull roof, above the posterior half of the orbit (Fig. 3A). No midline suture is visible (Figs. 1 and 3C). It has sutural contact with the frontal rostrally and the parietal caudally (Fig. 1 and 3). Its posterior margin is slightly elevated and contributes to the pineal boss and the anterior margin of the pineal foramen (Figs. 3A and 3C).

In lateral view the postfrontal is a prominent bone that has a vertical orientation (Figs. 1A, 1B and 3). This vertical orientation might be exaggerated by lateral compression. The postfrontal sutures with the frontal anteromedially, the parietal posteromedially, and the postorbital posterolaterally (Fig. 3).

The paired parietal bones are separated by a distinct midline suture (Fig. 3C) and the chimney-like pineal foramen is completely included within the parietals (Fig. 3C). Because of compression, the sutural contacts of the parietal are unclear on the left side. However, it is evident that the parietal contacts the preparietal anterolateral to the pineal foramen through a tripartite connection between the frontal, preparietal, and the parietal. Laterally, the parietal contacts the postfrontal, and extends anteriorly to form another tripartite suture between the postfrontal, the frontal, and parietal (Figs. 3C and 3D). Finally, the lateral margin of the parietal is overlapped by the postorbital (Figs. 3D and 3F), with a small posterolateral cleavage above the postorbital (Fig. 3F).

The right jugal of RC 55 forms the posterior half of the ventral and the posteroventral margins of the orbit (Figs. 1 and 2). It is an anteroposteriorly elongated bone that contacts the lacrimal and the maxilla anteriorly (Figs. 2A, 2B and 2F), and the postorbital posteriorly (Fig. 1A). The suture with the squamosal is unclear because of poor preservation (Fig. 1A). The left jugal has been displaced vertically and rotated counter clockwise such that the dorsal portion of the displaced bone is actually the posterior portion of the jugal (Fig. 2B). Its two extremities are vertically flat and V-shaped for articulation with the maxilla anteriorly and the squamosal posteriorly. This gives it a double-wrench overall outline. Medially, the long shaft of the jugal is slightly concave for insertion of the masseter muscle.

The postorbital is a T-shaped bone. Its ventral extension forms the postorbital bar and marks the posterior margin of the orbit and the anterior border of the temporal fenestra (Figs. 1 and 3), but the nature of the contact with the squamosal and jugal is uncertain (Fig. 1A). The postorbital contacts the squamosal dorsomedially and forms the dorsal and posterior margin of the temporal fenestra in lateral view (Figs. 1B and 1C). Anterodorsally the postorbital contacts the postfrontal and frontal; and the parietal posterodorsally (Fig. 3). Medially, the ventral process of the postorbital has a caudally curved ridge that extends from the postfrontal/postorbital suture to the possible jugal/postorbital contact (Figs. 1A, 1B and 3B).

**Figure 4:**
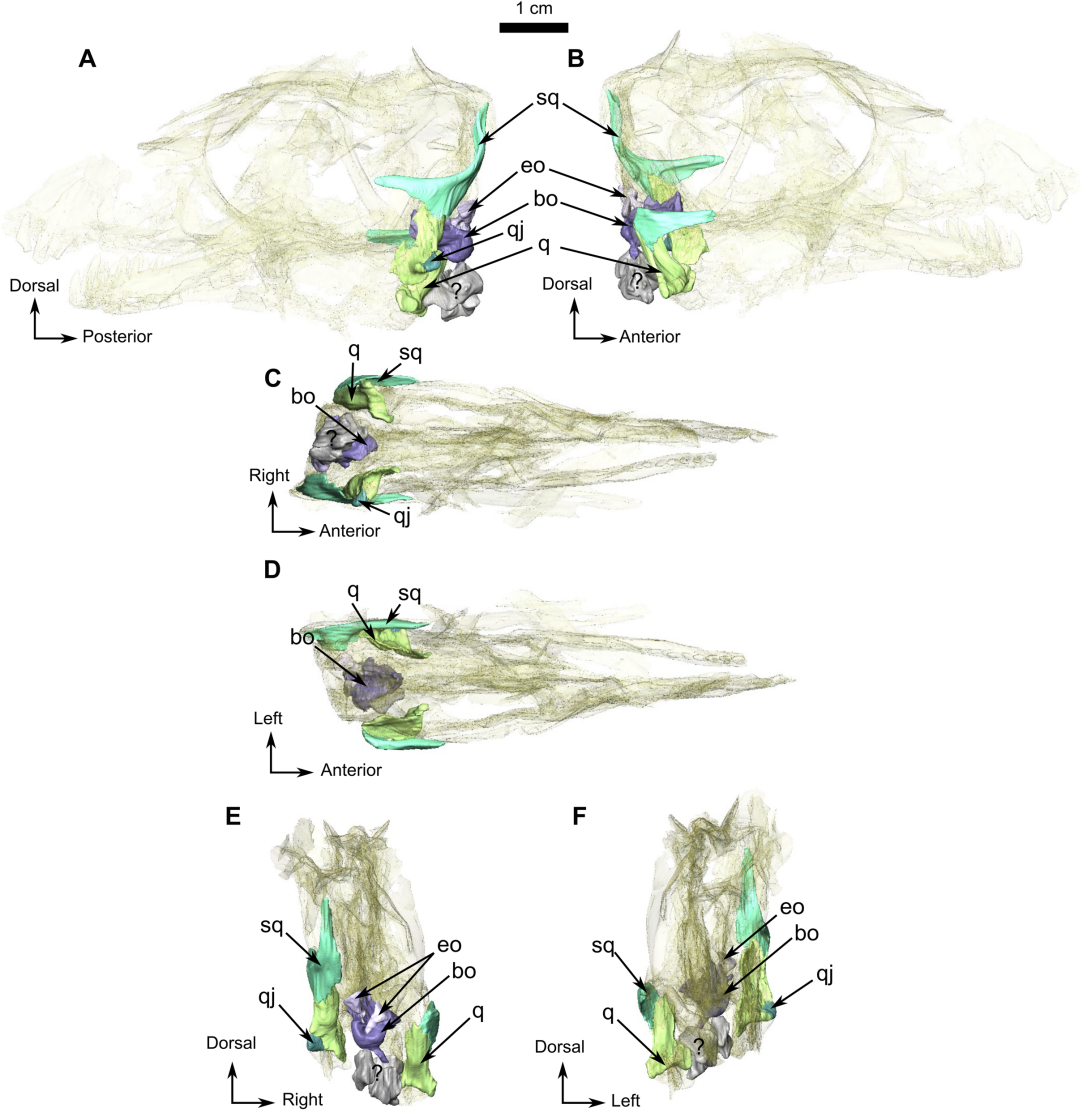
Holotype of *Rubidgina angusticeps*, here considered Biarmosuchia indet., RC 55. 3D rendering of the occipital portion of the skull of RC 55. (A) Left lateral view. (B) Right lateral view. (C) Ventral view. (D) Dorsal view. (E) Occipital view. (F) Anterior view. Anatomical Abbreviations—ang, angular; art, articular; aso, anterior extension of the supraoccipital; bo, basioccipital; c, caniniform tooth; co, occipital condyle; cor, coronoid; d, dentary; ect, ectopterygoid; eo, exoccipital; f, frontal; fe, temporal fenestra; fm, foramen magnum; i, incisiform tooth; j, jugal; l, lacrimal; m, maxilla; n, nasal; o, orbit; op, opisthotic; p, parietal; pa, anterior portion of the parietal; pac, caudal portion of the parietal; pal, palatine; pal t, palate teeth; pao, paroccipital process; part, pre-articular; pbs, parabasisphenoid; pc, postcanine; pf, postfrontal; pif, pineal foramen; pl, lateral portion of the parietal; pm, premaxilla; po, postorbital; pp, postparietal; prf, prefrontal; pro, prootic; prp, preparietal; pt, pterygoids; ptf, post-temporal fenestra; q, quadrate; qj, quadratojugal; rt, replacement tooth; sb, supraorbital boss; scl, sclerotic ring; smx, septomaxilla; so, supraoccipital; spl, splenial; st, stapes; sq, squamosal; sur surangular; t, tabular; v, vomer. 3D reconstructions were made by A. Duhamel and belong to the University of the Witwatersrand. Scale bar = 1 cm.

The squamosal is a crescent-shaped bone forming the posterior and ventral margins of the temporal fenestra (Fig. 4A) and is best preserved on the left side. It possibly contacts the postorbital on the zygomatic arch below the anterior margin of the temporal fenestra (Fig. 1A). In occipital view, the squamosal overlies the quadrate dorsally (Figs. 4A, 4B, 4E, and 4F). In medial view (Fig. 4B), the squamosal cradles the posterior side of the dorsal process of the quadrate. Anteroventrally, the squamosal has a triangular fossa at the base of the zygomatic process (Fig. 4B), which may represent the facet for the now-displaced jugal.

Both quadrates of RC 55 are well preserved (Fig. 4). This bone is vertically flattened and looks almost rectangular in occipital view. It is positioned on the anteromedial side of the squamosal (Figs. 4C and 4D). In occipital view (Fig. 4E) the ventral articulatory margin of the quadrate, comprising the lateral and the medial condyles, slopes ventrolaterally and has a smooth surface that articulates with the articular (Figs. 1C, 1E and 4E). Dorsal to the lateral condyle, the quadrate has a small fossa (Fig. 4F) where it contacts the small quadratojugal. On the right side, the quadrate appears to share a contact with the angular (Figs. 1A).

The quadratojugal is a small ovoid bone surfacing on the lateral side of the quadrate (Figs. 4A and 4E) and, like in other synapsids, participates in the jaw articulation with the quadrate on the skull, and the articular and the angular on the lower jaw (Figs. 1A, 1B, and 1E). This tiny quadratojugal constitutes the dorsolateral part of the jaw articulation. Only the left quadratojugal is preserved (Fig. 4), and it articulates solely with the quadrate on its dorsal, medial, and ventral borders (Figs. 4E and 4F).

### Occiput and basicranium

The occiput has a roughened surface and lateral compression has resulted in the occiput having a square outline in posterior view. The occipital surface is poorly preserved, most of the bones are crushed and could not be differentiated during segmentation (Figs. 1C and 1E). However, the jaw articulation is complete and well preserved in RC 55.

The small triangular exoccipital bone is preserved lateral to the foramen magnum (Figs. 4B and 4E) and its medial margin meets the basioccipital (Figs. 4B and 4E).

The basioccipital, which is roughly cylindrical, is poorly preserved (Fig. 4). The condyle is rounded in occipital view and extends ventrolaterally into a broken basioccipital tubera (Figs. 4E and 4F). In dorsal view, the basioccipital is concave medially, probably for the pituitary fossa (Fig. 4D), but because of poor preservation this could not be confirmed from the CT data. In ventral view, a midline ridge extends from the middle of the basioccipital to the anterior border of the occipital condyle (Fig. 4C).

Three unidentified bones are positioned ventral to the basioccipital (Figs. 4A, 4E and 4F). Although they cannot be identified with certainty, they may represent fragments of the prootic, opisthotic, basioccipital tubera, and/or stapes.

### Palate

The palate has been damaged as a result of lateral compression. The vomers and the ectopterygoids are missing, and some sutures are unclear, especially the one between the parabasisphenoid and pterygoid (Fig. 1E). Because of their fragmentary nature (Fig. 1E), it was not possible to digitally segment and isolate the posteriormost palatal bones and those of the basicranium, such as the parabasisphenoid. The right side of the palate is best preserved and is thus the basis of this description.

**Figure 5:**
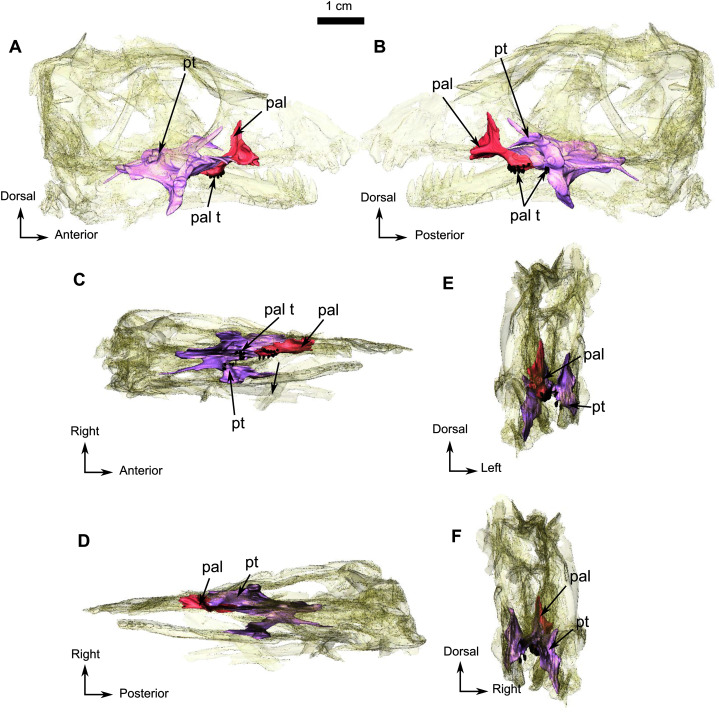
Holotype of *Rubidgina angusticeps*, here considered Biarmosuchia indet., RC 55. 3D rendering of the palatine portion of the skull of RC 55. (A) Right lateral view. (B) Left lateral view. (C) Ventral view. (D) Dorsal view. (E) Anterior view. (F) Occipital view. (G) Ventral view with lower jaw. (H) Dorsal view with lower jaw. Anatomical Abbreviations—ang, angular; art, articular; aso, anterior extension of the supraoccipital; bo, basioccipital; c, caniniform tooth; co, occipital condyle; cor, coronoid; d, dentary; ect, ectopterygoid; eo, exoccipital; f, frontal; fe, temporal fenestra; fm, foramen magnum; i, incisiform tooth; j, jugal; l, lacrimal; m, maxilla; n, nasal; o, orbit; op, opisthotic; p, parietal; pa, anterior portion of the parietal; pac, caudal portion of the parietal; pal, palatine; pal t, palate teeth; pao, paroccipital process; part, pre-articular; pbs, parabasisphenoid; pc, postcanine; pf, postfrontal; pif, pineal foramen; pl, lateral portion of the parietal; pm, premaxilla; po, postorbital; pp, postparietal; prf, prefrontal; pro, prootic; prp, preparietal; pt, pterygoids; ptf, post-temporal fenestra; q, quadrate; qj, quadratojugal; rt, replacement tooth; sb, supraorbital boss; scl, sclerotic ring; smx, septomaxilla; so, supraoccipital; spl, splenial; st, stapes; sq, squamosal; sur surangular; t, tabular; v, vomer. 3D reconstructions were made by A. Duhamel and belong to the University of the Witwatersrand. Scale bar = 1 cm.

The anterior extension of the palatine extends posteriorly along the alveolar margin from the last postcanine to the posterior margin of the palatine boss (Fig. 5C), which in turn is in contact with the pterygoid boss. Only the right palatine is preserved in RC 55 and contacts the maxilla anteriorly and anterolaterally (Fig. 1B), and the pterygoid posteriorly and posterolaterally (Fig. 5). The dentigerous palatine boss bears eleven teeth arranged in a single U-shaped row. In medial view (Fig. 5B), the palatine has a dorsal process that projects vertically from the anterior ramus of the palatine (Figs. 1B and 5B) and reaches the level of the dorsal margin of the maxilla (Fig. 1B) to form the posteroventral margin of the maxillary sinus. A small vertical lamina present on the palatine also contributes to the lateral wall of the sinus. In lateral view, the ascending process sutures with the maxilla and is excavated by an ovoid concavity (Fig. 5A). Medially and ventrally, the palatine shares a diagonal suture with the pterygoid along the lateral margin of the palatine boss (Figs. 5B and 5C).

In all Biarmosuchia, the pterygoid is a tripartite, paired bone, comprising the anteromedial process (or pterygoid *corpus*), the transverse process, and the quadrate ramus, the last of which forms the posterolateral part of the pterygoid (Rubidge & Sidor, 2002; Sidor & Welman, 2003; Rubidge & Kitching, 2003; Sidor, Hopson & Keyser, 2004; Kammerer, 2016; Day et al., 2018). On RC 55, the palatine ramus expands anteriorly between the maxilla laterally and the palatine medially (Fig. 5C). A low longitudinal ridge is present on the lateral margin of the palatine ramus (Fig. 5C). A high vertical lamina extends anterodorsally from the dorsal surface of the palatine ramus and reaches the level of the posteromedial border of the lacrimal dorsally (Figs. 1B and 5B). Posteromedial to the palatine ramus, the right pterygoid boss bears six teeth which are circular in cross-section. The right transverse process forms a 90° angle with the rest of the pterygoid in ventral view (Fig. 5C) and three palatal teeth are preserved on the left transverse process but there are none on the right. A posteromedially oriented ridge marks the transition between the transverse process and the quadrate ramus (Fig. 5C). The latter is divided into a thick posteromedial process and a thinner posterolateral process which is directed toward the quadrate (Fig. 5F).

### Lower jaw

**Figure 6:**
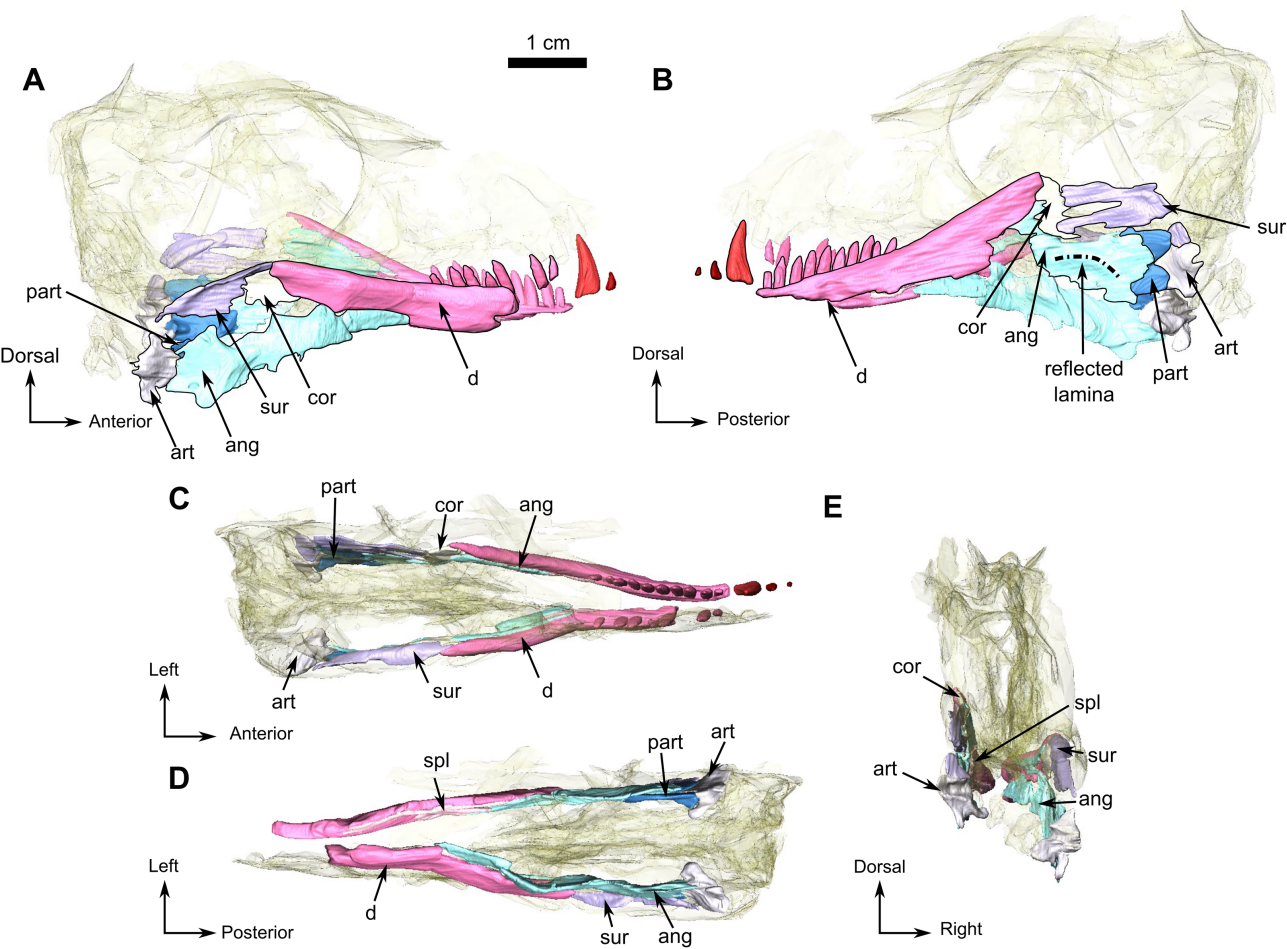
Holotype of *Rubidgina angusticeps*, here considered Biarmosuchia indet., RC 55. 3D rendering of the lower jaw of RC 55. (A) Right lateral view. (B) Left lateral view. (C) Dorsal view. (D) Ventral view. (E) Occipital view. Black outlines denote bones situated in the first plan of the figure. Anatomical Abbreviations—ang, angular; art, articular; aso, anterior extension of the supraoccipital; bo, basioccipital; c, caniniform tooth; co, occipital condyle; cor, coronoid; d, dentary; ect, ectopterygoid; eo, exoccipital; f, frontal; fe, temporal fenestra; fm, foramen magnum; i, incisiform tooth; j, jugal; l, lacrimal; m, maxilla; n, nasal; o, orbit; op, opisthotic; p, parietal; pa, anterior portion of the parietal; pac, caudal portion of the parietal; pal, palatine; pal t, palate teeth; pao, paroccipital process; part, pre-articular; pbs, parabasisphenoid; pc, postcanine; pf, postfrontal; pif, pineal foramen; pl, lateral portion of the parietal; pm, premaxilla; po, postorbital; pp, postparietal; prf, prefrontal; pro, prootic; prp, preparietal; pt, pterygoids; ptf, post-temporal fenestra; q, quadrate; qj, quadratojugal; rt, replacement tooth; sb, supraorbital boss; scl, sclerotic ring; smx, septomaxilla; so, supraoccipital; spl, splenial; st, stapes; sq, squamosal; sur surangular; t, tabular; v, vomer. 3D reconstructions were made by A. Duhamel and belong to the University of the Witwatersrand. Scale bar = 1 cm.

Both mandibular rami are damaged anterior to the level of the canine (Figs. 1 and 6) and the right ramus is missing the canine and incisors, but the canine and two incisors are preserved on the left (Fig. 1).

Only the posterior part of the dentary is preserved in RC 55. It is a long and thin bone on the dorsolabial surface of the lower jaw and has ten tooth sockets on the left and five on the right ramus (Figs. 6A and 6B). The dorsal margin of the dentary thickens posterodorsally to form a low coronoid process at the highest point of the lower jaw, where the dentary, the coronoid and the surangular meet (Fig. 6B). Posteromedially, the dentary has a straight vertical contact with the prearticular and the splenial (Figs. 6A, 6B and 6D).

The splenial is a delicate elongated bone on the medial side of the dentary and is not visible in lateral view (Fig. 6). Posterodorsally it has a sutural contact with the angular (Fig. 6D) and anteriorly it is broken at the level of the 9^th^ postcanine.

A small coronoid is positioned on the dorsal part of both lower jaw rami (Fig. 6) and is present between the dentary anteriorly and the surangular posteriorly (Figs. 6-A and B). On the left lower jaw ramus, the coronoid cradles the anterior margin of the upper mandibular fenestra (Fig. 6-B).

The angular is a large flat bone that forms a third of the posteroventral part of the lower jaw on the medial side (Figs. 6A and 6B) and is damaged along its ventral edge as a result of post-mortem weathering (Figs. 6A and 6B). The elongated and thin anterior portion is positioned ventromedial to the dentary (Fig. 6A). This portion of the angular contacts the splenial anteriorly and the coronoid and the surangular dorsally (Figs. 6A and 6B). The posterior portion of the angular is broad with a damaged posteroventral margin (Figs. 6A and 6B) and contacts the prearticular posteriorly. Postmortem damage resulted in an opening between the angular and the surangular on the left ramus (Fig. 6B). Ventral to this opening the angular bears the reflected lamina that is preserved on the left ramus (Fig. 6B). The reflected lamina of RC 55 consists of a thin ridge curving dorsally, that extends along the the ventral margin of the angular (Fig. 6B). As this ventral margin has been weathered away, it is possible that the ventral-most part of the reflected lamina is not preserved on RC 55.

The poorly preserved surangular makes up the posterodorsal part of the lower jaw, posterior to the coronoid, anterior to the angular, and dorsal to the prearticular (Fig. 6B). The surangular of RC 55 is badly preserved, however it is clear that the surangular forms most of the dorsal margin of an opening, bordered anteriorly by the coronoid, and is due to possible post-mortem damages (Fig. 6B).

The prearticular is a small bone of the lower jaw located posteriorly, and exposed mainly on the medial side of both rami (Figs. 6A and 6D). It is better preserved on the left ramus and surrounds the anterior margin of the articular posteriorly (Fig. 6B), while sharing only a short contact with the articular on the right ramus (Fig. 6A). The prearticular contacts the surangular posterodorsally and the medial side of the angular anteriorly (Figs. 6A and 6D).

In RC 55, the articulation surface of the articular is concave and positioned posteromedially (Fig. 6D). In posterior view (Figs. 6D and 6E) the articular has two ventral processes separated by a deep notch (Figs. 6D and 6E).

### Dentition

**Figure 7:**
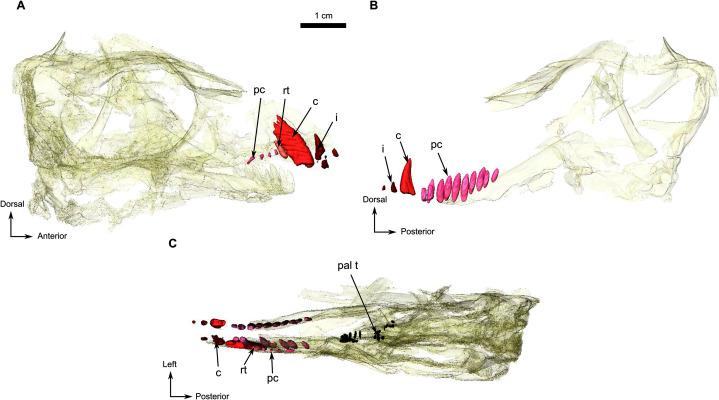
Holotype of *Rubidgina angusticeps*, here considered Biarmosuchia indet., RC 55. 3D rendering of the dentition of RC 55. (A) Right lateral view. (B) Left lateral view. Anatomical Abbreviations—ang, angular; art, articular; aso, anterior extension of the supraoccipital; bo, basioccipital; c, caniniform tooth; co, occipital condyle; cor, coronoid; d, dentary; ect, ectopterygoid; eo, exoccipital; f, frontal; fe, temporal fenestra; fm, foramen magnum; i, incisiform tooth; j, jugal; l, lacrimal; m, maxilla; n, nasal; o, orbit; op, opisthotic; p, parietal; pa, anterior portion of the parietal; pac, caudal portion of the parietal; pal, palatine; pal t, palate teeth; pao, paroccipital process; part, pre-articular; pbs, parabasisphenoid; pc, postcanine; pf, postfrontal; pif, pineal foramen; pl, lateral portion of the parietal; pm, premaxilla; po, postorbital; pp, postparietal; prf, prefrontal; pro, prootic; prp, preparietal; pt, pterygoids; ptf, post-temporal fenestra; q, quadrate; qj, quadratojugal; rt, replacement tooth; sb, supraorbital boss; scl, sclerotic ring; smx, septomaxilla; so, supraoccipital; spl, splenial; st, stapes; sq, squamosal; sur surangular; t, tabular; v, vomer. 3D reconstructions were made by A. Duhamel and belong to the University of the Witwatersrand. Scale bar = 1 cm.

The anterior dentition of RC 55 is not preserved because of incomplete preservation of the anterior end of the lower jaws and snout (Figs. 1 and 7). Despite this, CT imagery has, for the first time, revealed the presence of three small upper and two lower incisors (Fig. 7). The last upper incisor seems to have a caniniform morphology but is too poorly preserved to ascertain. One sharp caniniform is present on the lower jaw, and the upper canine has suffered post-mortem deformation. As in SAM-PK-K11126, a replacement upper canine is positioned posterior to the functional one. The upper postcanines are small and are poorly preserved such that it is not possible to determine their morphology. In contrast, the lower jaw has ten well-preserved conical postcanines (Fig. 7).

The remaining palatine boss bears ten teeth which are circular in cross-section and arranged in a U-shaped single row (Figs. 1E and 5C). The six teeth on the pterygoid boss are smaller and randomly distributed. Three medially positioned teeth are present on the transverse process (Figs. 1E and 5C).

BIARMOSUCHIA Sigogneau-Russell, 1989

BURNETIAMORPHA Broom, 1923

BURNETIAMORPHA gen. et sp. indet.

**Material**–CGS MJF 22, an almost complete skull with the lower jaw. The anterior part of the snout and dentary are missing. It can be recognized as a burnetiamorph on the basis of a median ridge on the skull roof and a supraorbital boss; the palatal process of premaxilla is long and laterally bounds the anterior portion of vomer; and the surangular bears a deep fossa laterally. The genus cannot be determined because of its juvenile condition.

**Figure 8:**
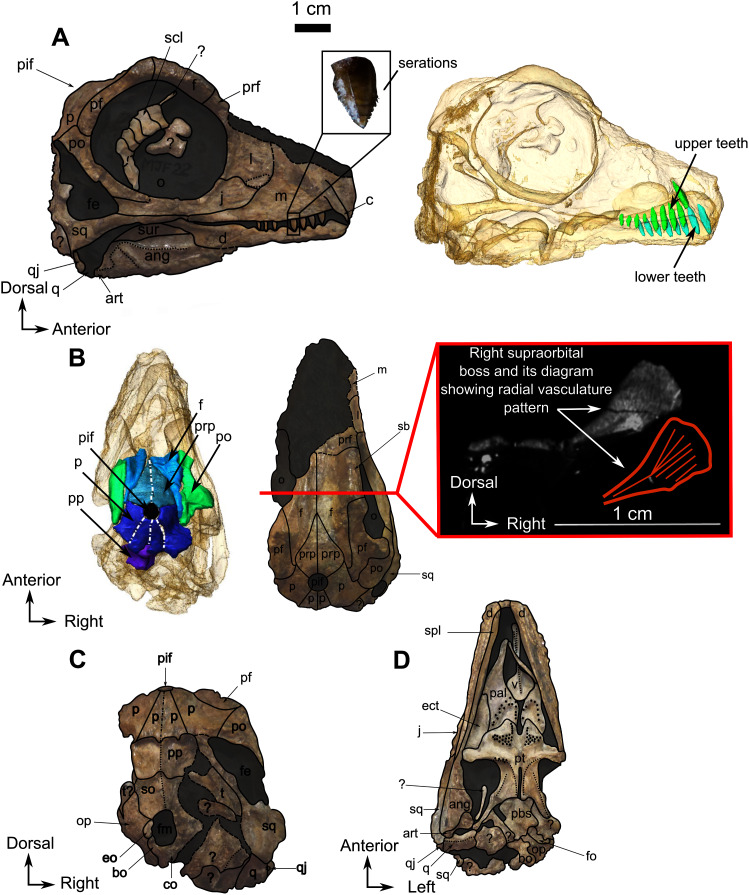
Burnetiamorpha indet., skull, CGS MJF 22. (A) Lateral view, with a 3D rendering of the teeth (right). (B) Dorsal view with the bones of the pineal region segmented on the left; and the CT image at the level of the supraorbital bosses and a diagram of the vasculature pattern found in the bosses (right). (C) Occipital view. (D) Ventral view. Questions mark denote unidentified bones. Short dotted line accentuates anatomical character; long dotted line indicates uncertainly about sutural pathway on bone. Anatomical Abbreviations—ang, angular; art, articular; aso, anterior extension of the supraoccipital; bo, basioccipital; c, caniniform tooth; co, occipital condyle; cor, coronoid; d, dentary; ect, ectopterygoid; eo, exoccipital; f, frontal; fe, temporal fenestra; fm, foramen magnum; i, incisiform tooth; j, jugal; l, lacrimal; m, maxilla; n, nasal; o, orbit; op, opisthotic; p, parietal; pa, anterior portion of the parietal; pac, caudal portion of the parietal; pal, palatine; pal t, palate teeth; pao, paroccipital process; part, pre-articular; pbs, parabasisphenoid; pc, postcanine; pf, postfrontal; pif, pineal foramen; pl, lateral portion of the parietal; pm, premaxilla; po, postorbital; pp, postparietal; prf, prefrontal; pro, prootic; prp, preparietal; pt, pterygoids; ptf, post-temporal fenestra; q, quadrate; qj, quadratojugal; rt, replacement tooth; sb, supraorbital boss; scl, sclerotic ring; smx, septomaxilla; so, supraoccipital; spl, splenial; st, stapes; sq, squamosal; sur surangular; t, tabular; v, vomer. Pictures by A. Duhamel. Scan of the specimen was performed by Kudakwashe Jakata and belongs to the University of the Witwatersrand. 3D reconstructions were made by A. Duhamel and belong to the University of the Witwatersrand. Scale bar = 1 cm.

**Description**–Specimen CGS MJF 22 (Fig. 8) comprises an almost complete skull and occluded lower jaw. On specimen CGS MJF 22, the roof of the snout and part of the left side are damaged. Superficially the skull is broadly triangular in lateral view (Fig. 8A). The right side of the skull is relatively well preserved, allowing most bones to be digitally segmented (Fig. 8). Similarly, the right ramus of the lower jaw is well-preserved except for the anterior tip of the dentary, which is missing. The left lower jaw is badly weathered and preserves only the anterior portion of the dentary. The large orbit makes up about 70% of the lateral surface of the skull (orbit diameter is 30 mm and basal skull length is 77 mm). It forms an almost perfect circle and at least six sclerotic plates from the posterodorsal region of the sclerotic ring are preserved in the right orbit. As the left side is poorly preserved, this description is based mostly on the right side. The palate is well preserved and much of the anatomy is visible, including the palatal teeth. The medial portion of the occiput is well preserved, but the left side is missing and the right side is weathered, which prevents accurate description.

### Skull

The anterior tip of the skull is weathered away, and it is not possible to determine the morphology of the premaxilla, the septomaxilla, and the arrangement of the external nares. The right lateral surface of the maxilla is well preserved. It is a smooth and triangular bone (Fig. 8A) that covers most of the lateral surface of the snout. The long, straight alveolar margin bears one canine and eight postcanines, the last of which is unerupted and has been revealed by CT (Fig. 8A). Along its posterior margin, the maxilla shares a suture with the lacrimal and sends out a thin strip-like posterior process that tapers below the jugal ventrally. This process probably shares a short contact with the squamosal on the zygomatic arch below the orbit, but the contact is not visible.

The lacrimal is a quadrangular bone, forming most of the anterior margin of the orbit (Fig. 8A) and contacts the jugal ventrally, the maxilla anteriorly, and the prefrontal dorsally but the anterior part of the prefrontal is not preserved. A vertical ridge extends up the lacrimal along the anterior border of the orbit. The lacrimal is excavated by an ovoid depression anterior to the orbit, close to the suture between the maxilla and jugal.

The jugal comprises almost the entire ventral margin of the orbit and forms a small part of the anteroventral margin of the temporal fenestra (Fig. 8A). Anterodorsally it shares a horizontal sutural contact with the lacrimal and meets the maxilla anteroventrally, the squamosal posteroventrally, and the postorbital posterodorsally. The suborbital portion of the zygomatic arch formed by the jugal is thin and not pachyostosed. The small triangular temporal fenestra is less than one third the size of the orbit and is positioned posteroventral to the orbit. It is bordered anteriorly by the postorbital and the jugal, and posteriorly by the squamosal.

The narrow postorbital bone slopes posterodorsally at an angle of 45° to the horizontal and forms practically the entire postorbital bar (Fig. 8A). Anterodorsally it meets the postfrontal at the posterior margin of the orbit, has a long sutural contact with the postfrontal on the skull roof, and meets the parietal posteromedially such that the postorbital has extensive exposure on the skull roof (Fig. 8C).

The squamosal is a comparatively large bone forming the ventral and posterior borders of the temporal fenestra. It shares a suture with the tabular on the occiput, and ventrally it contacts the quadrate and quadratojugal (Figs. 8A and 8C). Anteriorly, the squamosal tapers into a long process that sutures with the posterior process of the maxilla (Figs. 8A and 8C).

As many of the skull roof bones are slightly pachyostosed (Fig. 8B), their sutural contacts are not readily visible and were identified using CT data. The orbital rim bears a conspicuous supraorbital boss that reaches its highest point halfway above the orbit and is formed mainly by the frontal and the postfrontal. A large domed boss surrounds the circular pineal foramen (Fig. 8B) which is positioned posteromedial to the orbit. The pineal tube extends anteroventrally at an angle of about 70° relative to the skull axis (Fig. 8A). Only a small portion of the posterior end of the right prefrontal is preserved. It constitutes the anterior-most part of the supraorbital boss and forms the anterodorsal rim of the orbit (Fig. 8B). Although the degree of pachyostosis of the supraorbital boss is low in CGS MJF 22, the CT data revealed the presence of radial structures in coronal cross-section (Fig. 8B), which are considered to be radial vascular structures linked to the possibly juvenile status of the specimen (see “Discussion”).

The paired frontals have a smooth dorsal surface with no midline ridge (Fig. 8B). Each frontal contributes a large portion of the supraorbital part of the skull roof, participates in the dorsal margin of the orbit, and tapers posterolaterally between the preparietal and postfrontal. The posterior tip of the frontal forms a pointed contact with the parietal at the level of the anterior margin of the pineal foramen.

The preparietal is a relatively large, roughly triangular, paired bone positioned between the two supraorbital bosses (Fig. 8B) with the apex of the preparietal tapering anteriorly between the frontals. Posteromedially, the preparietal contributes to the border of the pineal foramen, and posterolaterally it shares an oblique sutural contact with the parietal. A midline suture is present in the preparietal (Fig. 8).

The parietal contributes to the lateral margin of the pineal foramen and extends onto the lateral and posterior sides of the pineal boss (Fig. 8B). In dorsal view, it contacts the preparietal anteromedially, shares a short contact with the frontal and postfrontal anterolaterally, with the postorbital posterolaterally, and the postparietal posteriorly on the occiput (Fig. 8C). In addition, the postfrontal forms a posteriorly directed process between the frontal and the postorbital. Based on the CT images it appears that the parietal is divided into two separate bones (Fig. 8B), a rostrolateral part and a posteromedial part radiating posteriorly from the pineal foramen (Fig. 8), which may indicate two distinct centres of ossification (see “Discussion”). The midline suture between the paired parietals, posterior to the pineal foramen, is not fused (Fig. 8).

Most of the left side of the occiput of CGS MJF 22 is missing and the right side is badly weathered (Fig. 8C). However, the central portion is preserved. The postparietal is a large unpaired rectangular bone that forms the dorsal half of the occiput. Ventrally it shares a horizontal suture with the supraoccipital and touches the parietal dorsally. Because of extensive post-mortem damage, the lateral suture with the tabular is impossible to determine. However, a short dorsolateral contact with the right squamosal is evident. A vertical midline ridge, the external occipital ridge, extends ventrally from the postparietal to the foramen magnum (Fig. 8C) and is more rounded on the supraoccipital than on the postparietal.

The unpaired supraoccipital is not well preserved, and the left side is the most complete (Fig. 8C). It forms the dorsal margin of the foramen magnum and extends laterally and ventrally to contact the exoccipital ventrally, and the opisthotic ventrolaterally. Dorsally it shares a long horizontal contact with the postparietal.

The opisthotic is mostly weathered, its exact shape cannot be determined, and it is only partly preserved on the left side of the occiput (Fig. 8C). It contacts the supraoccipital dorsomedially, the exoccipital medially, and the basioccipital ventromedially. On the ventral side (Fig. 8D), the opisthotic is oriented posterolaterally and shares a sinusoidal medial contact with the basioccipital. The opisthotic contacts the parabasisphenoid anteromedially, and bears the fenestra ovalis anteriorly. This fenestra is partially crushed laterally and has a straight sutural contact with the opisthotic medially (Fig. 8D).

A small, oval exoccipital forms the lateral border of the foramen magnum (Fig. 8C). Ventrally, it contacts the basioccipital, dorsally the supraoccipital, and the opisthotic laterally. The right exoccipital is not preserved.

The midline basioccipital forms the ventral and ventrolateral margin of the foramen magnum and the occipital condyle (Fig. 8C). It contacts the exoccipital dorsolaterally and the opisthotic laterally. Only a small portion of the left basioccipital condyle is preserved ventrally. In palatal view (Fig. 8D), the basioccipital contacts the opisthotic laterally and the parabasisphenoid anteriorly.

### Palate

Generally, the ventral side of the skull is well preserved except for the posterior part close to the basicranium (Fig. 8D). Anteriorly, the vomer is a thin midline bone which has not been extensively prepared because it is too delicate. From the CT data, it appears that the vomer is an unpaired structure with a medial trough flanked by two thin vertically oriented lateral flanges. Posteriorly, the vomer forms a large expanded vomerine plate (Fig. 8D). The lateral flanges of the vomers converge posteriorly to form a midline ridge that extends posteriorly onto the vomerine plate (Fig. 8D) which has posterior contact with the palatines. The horizontal suture between the vomerine plate and the palatine is more dorsally positioned than the rest of the palate.

The palatine and pterygoid form most of the palate (Fig. 8D). The palatine bears a long anterior tongue-like process that forms the lateral margin of the internal naris and extends anteriorly to the level of the caniniform tooth. A prominent palatine boss protrudes ventrally and bears numerous nubbin-like teeth arranged in a U-shaped pattern (Fig. 8D). Twenty small teeth are present on the right palatine boss and are arranged in two rows (Fig. 8D). The left palatine boss is not well preserved. A midline trough separates the paired palatine and pterygoid bosses. Posteriorly, the palatine shares a reverse V-shaped suture with the pterygoid and meets the ectopterygoid posterolaterally (Fig. 8D).

The ectopterygoid (Fig. 8D) is preserved on the right side. It is an anteroposteriorly long and flat bone that constitutes the lateral aspect of the palate. Anteriorly and anteromedially it has a long sutural contact with the palatine and posteriorly contacts the pterygoid.

The tripartite paired pterygoid comprises the pterygoid boss anteriorly, the transverse process, and the quadrate ramus caudally (Fig. 8D). The pterygoid boss is in continuity with the palatine boss and also bears small nubbin-like teeth (Fig. 8D). On the right pterygoid boss, about 25 teeth are clustered randomly, but because of the poor preservation of the left side, the exact number of teeth is uncertain (both on the specimen and the CT data). The transverse process is robust and positioned halfway along the pterygoid, at the level of the anterior margin of the orbit. On the medial side of the right transverse process, are four teeth arranged in a single row. As a result of post-mortem damage, it is not possible to determine whether teeth are present on the left transverse process and metallic inclusions prevent clear identification on the CT data. Posterior to the transverse process, the pterygoid *corpus* is excavated by a long slit-like interpterygoid vacuity (Fig. 8D). The raised margins of the interpterygoid vacuity are in continuity with the transverse processes. A long quadrate ramus extends back from the lateral side of the pterygoid *corpus*, posterior to the transverse process (Fig. 8D).

Posteromedially, the pterygoid *corpus* meets the parabasisphenoid (fusion of the parasphenoid and basisphenoid), which is slightly offset dorsally (Fig. 8D). On its ventral surface, this bone bears an elongated medial mound. Lateral to this mound, the parabasisphenoid forms a small shelf (Fig. 8D). Posterior to the mound, the suture with the basioccipital is unclear, but a very slight depression is present between the two basal tubera. Posterolaterally, the parabasisphenoid meets the opisthotic and forms the anteromedial border of the fenestra ovalis (Fig. 8D).

The quadrate forms the jaw articulation with the articular, and only the right quadrate is preserved (Fig. 8D). The ventral side of the quadrate is rectangular, mediolaterally elongated, and presents a medial crest. This crest is probably a result of the fusion between the two quadrate condyles as already observed in other biarmosuchian taxa (Sidor & Rubidge, 2006). The quadrate contacts the quadrate ramus of the pterygoid anteromedially and the quadratojugal posterodorsally (Fig. 8D). In lateral view, the quadrate has a small exposure at the posteroventral extremity of the skull (Fig. 8A), but in occipital view the dorsal process is broad and attaches to the anterior side of the squamosal ventral flange (Fig. 8C). It is in close contact with the articular such that its anterior aspect is not visible.

The quadratojugal is a small, thin, and vertically flat bone that is visible in lateral, occipital, and ventral views (Figs. 8A, 8C, 8D respectively) on the right side. On its lateral aspect, the rectangular quadratojugal contacts the quadrate dorsal to its lateral articular condyle (Fig. 8A). In occipital view the quadratojugal contacts the quadrate below the small quadratojugal foramen and the squamosal dorsally (Fig. 8C). In palatal view, the quadratojugal contacts the lateral quadrate condyle and has an articular contact with the angular anteriorly (Fig. 8D).

### Lower jaw

The right dentary is reasonably well preserved in comparison to the left, but the ventral surface has been weathered away. In lateral view, the dentary forms the anterior half of the mandible (Fig. 8A). Posteroventrally, the dentary contacts the angular with a posterodorsally oriented suture. The exact nature of the contact is unclear because of damage to the ventral part of the mandible from this point forward. The dentary meets the surangular and continues posterodorsally as a process that overlies the surangular and forms the dorsal margin of the mandible. Below the orbit, the dentary is mediolaterally compressed into a low coronoid process (Fig. 8A). The lateral surface of the dentary is relatively flat and smooth, as is typical in biarmosuchians (Rubidge & Kitching, 2003; Sidor, 2003; Sidor & Welman, 2003; Sidor, Hopson & Keyser, 2004; Rubidge, Sidor & Modesto, 2006; Smith, Rubidge & Sidor, 2006; Sidor & Smith, 2007; Kruger et al., 2015; Kammerer, 2016). The posterodorsal margin of the dentary forms a laterally projecting ridge that extends posteriorly, dorsal to the surangular. This structure originates dorsal to the contact of the dentary and angular, initially swelling into a round ridge but flaring caudally so that it is very thin as it projects over the surangular (Fig. 8A). The splenial is visible as a long, thin ribbon-like bone on the medial side of the jaw. It extends anteriorly from the ectopterygoid to the tip of the mandible (Fig. 8D).

In lateral view, the surangular has a small exposure on the posterodorsal side of the mandible, where it forms the curved posterodorsal edge of the coronoid process. It bears a prominent laterally projecting longitudinal ridge (Fig. 8A). On its ventral side, the surangular has a long horizontal sutural contact with the angular and contacts the dentary dorsally (Fig. 8D).

The angular forms the posterolateral portion of the lower jaw extending posteriorly from below the anterior margin of the orbit (Fig. 8A). The extensive reflected lamina bears a prominent oblique ridge that curves posterodorsally. In lateral view, the angular contacts the surangular dorsally and the dentary anteriorly (Fig. 8A). On the medial side it contacts the dentary anterolaterally and posteriorly reaches the two articular condyles (Fig. 8D). The anterior contact with the splenial cannot be determined because of poor preservation.

The poorly preserved articular is located posterior to the angular as is usual in Biarmosuchia (Sidor, Hopson & Keyser, 2004). Medially the articular bears two condyles, one lateral and one medial (Fig. 8D). The articular articulates with the quadrate posteriorly to form the jaw joint.

### Dentition

As the tip of the snout and mandible is eroded, no pre-canine teeth are preserved on the upper and lower jaw. On the maxilla, the weathered alveolus of the right caniniform tooth extends dorsally almost as far as the dorsal margin of the maxillary bone (Fig. 8A). CT data reveal the presence of an unerupted tooth posterior to the erupted caniniform socket that might be a replacement caniniform tooth (Fig. 8A). The scan also reveals eight marginal postcanines in the right maxilla, but no teeth are visible on the left side. The anterior-most three postcanines have serrations on their posterior edge, whereas the four posterior-most ones bear serrations on both the anterior and posterior edges (Fig. 8A). It was not possible to determine the serration pattern on the fourth postcanine.

No pre-canine or caniniform teeth are preserved on the lower jaw. Eight postcanines are visible on the right lower jaw (Fig. 8A). They all share a similar, conical morphology, and decrease in size posteriorly. No serrations are visible on the anterior-most post-canine tooth, but the second, third, and fourth teeth have serrations on the anterior side. The fifth post-canine tooth has serrations on both sides, the sixth does not have visible serrations, and the seventh and eighth post-canines have serrations on both sides. These seemingly random variations could be the result of over-preparation or differential weathering due do rapid dental replacement in this juvenile individual.

BIARMOSUCHIA Sigogneau-Russell, 1989

BURNETIAMORPHA Broom, 1923

cf. *Lophorhinus willodenensis* Sidor & Smith (2007)

**Material**–Specimen SAM-PK-K11126, a broken potential juvenile specimen which preserves a partial snout and palate, the pineal region, the occiput, a partial lower jaw, posterior dorsal and sacral vertebrae attached to a pelvis, a distal part of a femur and some indeterminate bones. SAM-PK-K11126 can be identified as a burnetiamorph because of the presence of a median ridge-like structure on the skull roof and a long palatal process of the premaxilla laterally bounding the anterior portion of the vomer. We think it likely referrable to *Lophorhinus willodenensis* because of the presence of a long palatal process of the premaxilla that laterally bounds the anterior part of the vomer (Sidor & Smith, 2007) and similarities in the shape of the vomer in SAM-PK-K11126 and SAM-PK-K6655 (holotype of *Lophorhinus willodenensis*).

**Figure 9:**
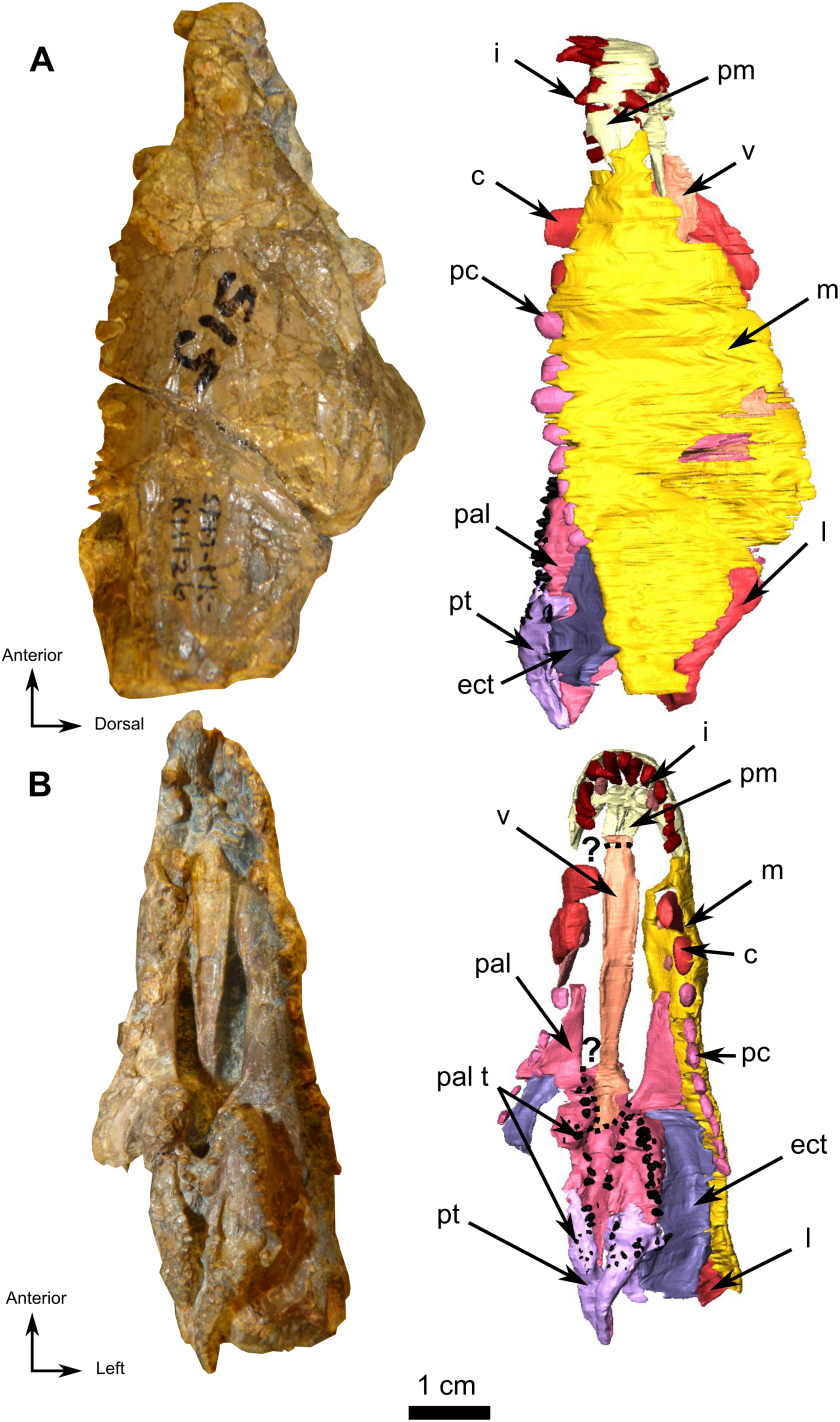
Burnetiamorpha cf. *Lophorhinus willodenensis*, snout, SAM-PK-K11126. Photograph (left) and 3D rendering of SAM-PK-K11126 (right). (A) Left lateral view. (B) Ventral view. Anatomical Abbreviations—ang, angular; art, articular; aso, anterior extension of the supraoccipital; bo, basioccipital; c, caniniform tooth; co, occipital condyle; cor, coronoid; d, dentary; ect, ectopterygoid; eo, exoccipital; f, frontal; fe, temporal fenestra; fm, foramen magnum; i, incisiform tooth; j, jugal; l, lacrimal; m, maxilla; n, nasal; o, orbit; op, opisthotic; p, parietal; pa, anterior portion of the parietal; pac, caudal portion of the parietal; pal, palatine; pal t, palate teeth; pao, paroccipital process; part, pre-articular; pbs, parabasisphenoid; pc, postcanine; pf, postfrontal; pif, pineal foramen; pl, lateral portion of the parietal; pm, premaxilla; po, postorbital; pp, postparietal; prf, prefrontal; pro, prootic; prp, preparietal; pt, pterygoids; ptf, post-temporal fenestra; q, quadrate; qj, quadratojugal; rt, replacement tooth; sb, supraorbital boss; scl, sclerotic ring; smx, septomaxilla; so, supraoccipital; spl, splenial; st, stapes; sq, squamosal; sur surangular; t, tabular; v, vomer. Vertical jagging on the 3D rendering is an artefact of segmentation. Pictures by A. Duhamel. 3D reconstructions were made by A. Duhamel and belong to the University of the Witwatersrand. Scale bar = 1 cm.

**Figure 10:**
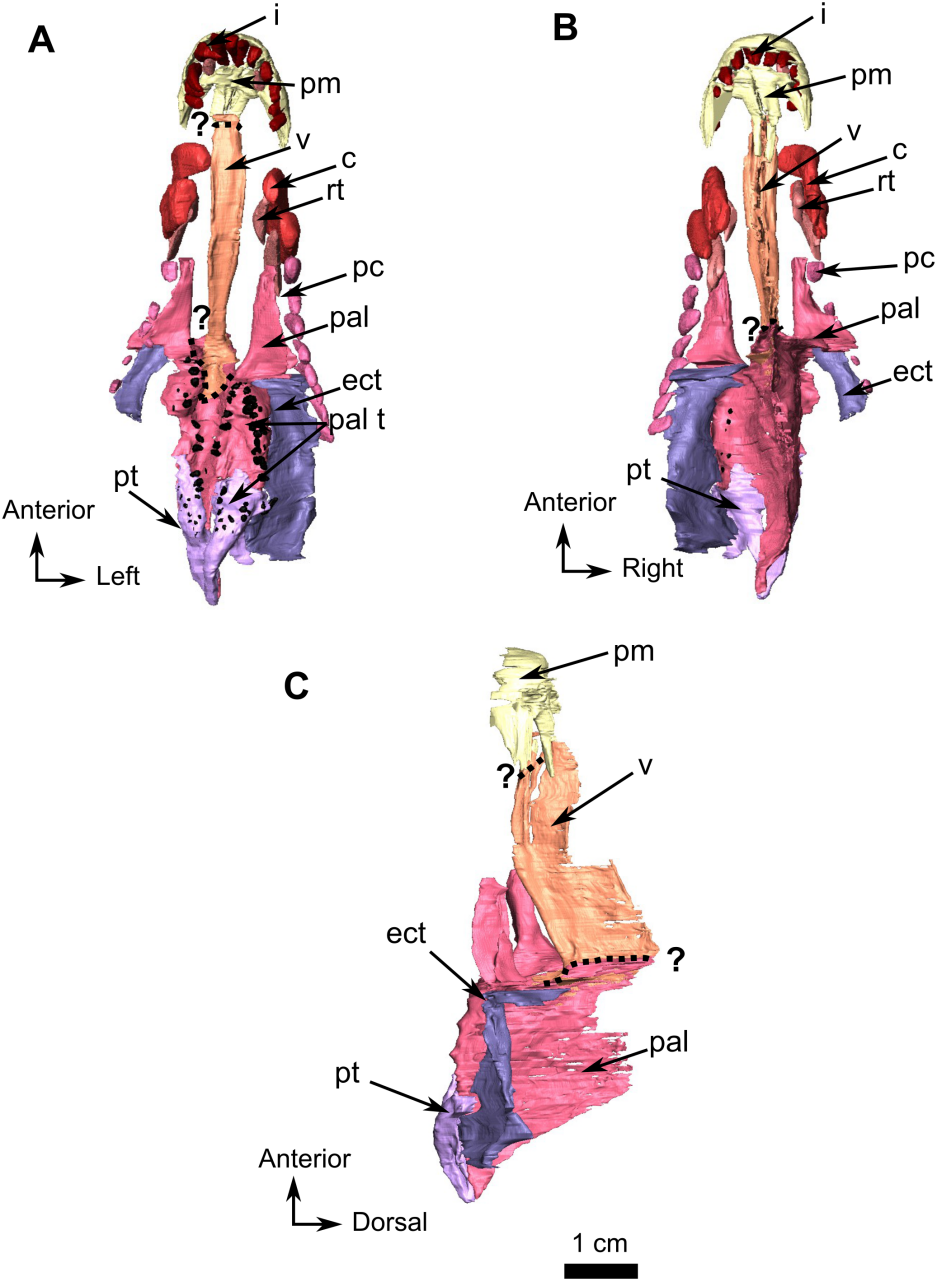
Burnetiamorpha cf. *Lophorhinus willodenensis*, palate, SAM-PK-K11126. 3D rendering of SAM-PK-K11126’s palatine bones and teeth (right). (A) Ventral view. (B) Dorsal view. (C) Left lateral view. Anatomical Abbreviations—ang, angular; art, articular; aso, anterior extension of the supraoccipital; bo, basioccipital; c, caniniform tooth; co, occipital condyle; cor, coronoid; d, dentary; ect, ectopterygoid; eo, exoccipital; f, frontal; fe, temporal fenestra; fm, foramen magnum; i, incisiform tooth; j, jugal; l, lacrimal; m, maxilla; n, nasal; o, orbit; op, opisthotic; p, parietal; pa, anterior portion of the parietal; pac, caudal portion of the parietal; pal, palatine; pal t, palate teeth; pao, paroccipital process; part, pre-articular; pbs, parabasisphenoid; pc, postcanine; pf, postfrontal; pif, pineal foramen; pl, lateral portion of the parietal; pm, premaxilla; po, postorbital; pp, postparietal; prf, prefrontal; pro, prootic; prp, preparietal; pt, pterygoids; ptf, post-temporal fenestra; q, quadrate; qj, quadratojugal; rt, replacement tooth; sb, supraorbital boss; scl, sclerotic ring; smx, septomaxilla; so, supraoccipital; spl, splenial; st, stapes; sq, squamosal; sur surangular; t, tabular; v, vomer. 3D reconstructions were made by A. Duhamel and belong to the University of the Witwatersrand. Scale bar = 1 cm.

**Figure 11:**
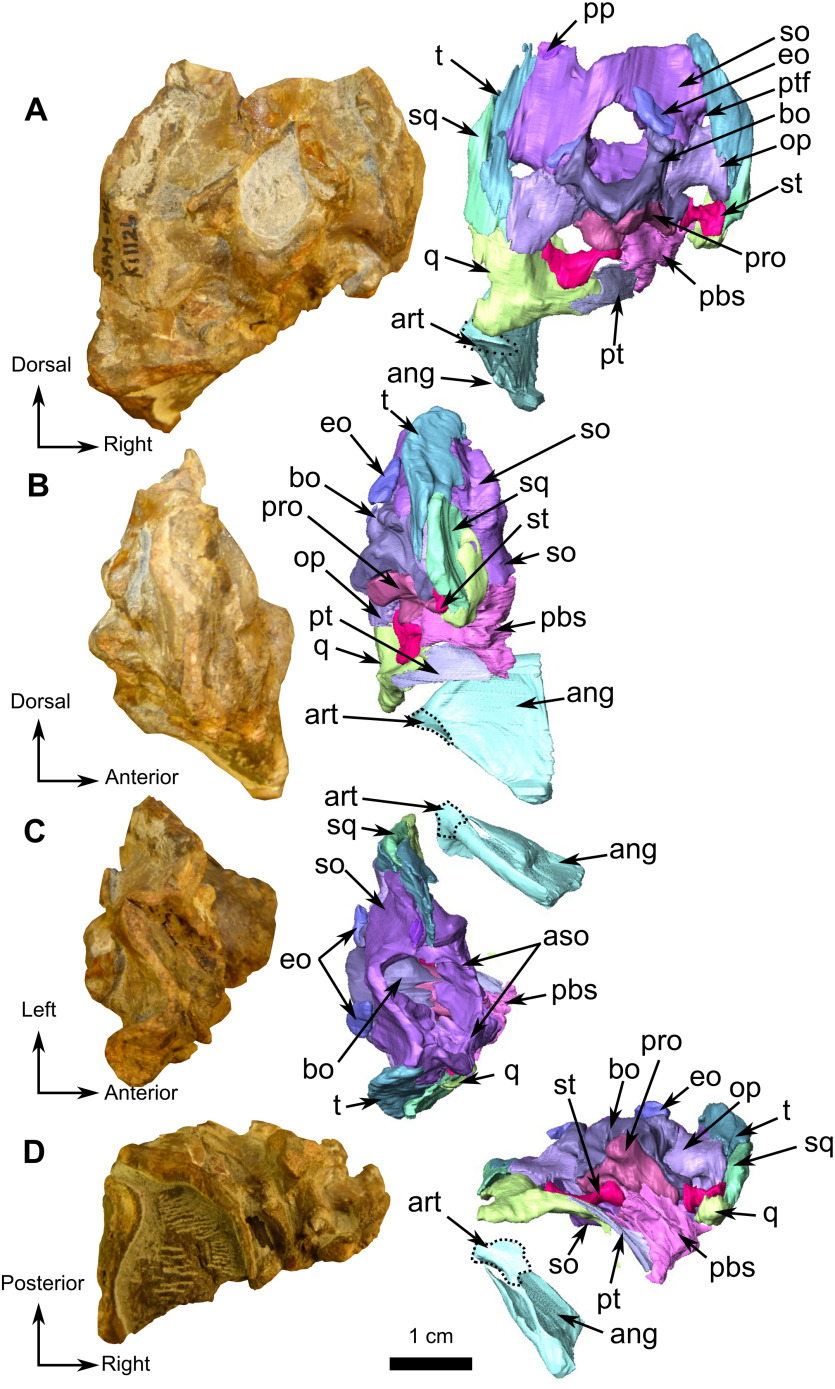
Burnetiamorpha cf. *Lophorhinus willodenensis*, occipital portion, SAM-PK-K11126. Photograph (left) and 3D rendering of SAM-PK-K11126 (right). (A) Occipital view. (B) Right lateral view. (C) Dorsal view. (D) Ventral view. Anatomical Abbreviations—ang, angular; art, articular; aso, anterior extension of the supraoccipital; bo, basioccipital; c, caniniform tooth; co, occipital condyle; cor, coronoid; d, dentary; ect, ectopterygoid; eo, exoccipital; f, frontal; fe, temporal fenestra; fm, foramen magnum; i, incisiform tooth; j, jugal; l, lacrimal; m, maxilla; n, nasal; o, orbit; op, opisthotic; p, parietal; pa, anterior portion of the parietal; pac, caudal portion of the parietal; pal, palatine; pal t, palate teeth; pao, paroccipital process; part, pre-articular; pbs, parabasisphenoid; pc, postcanine; pf, postfrontal; pif, pineal foramen; pl, lateral portion of the parietal; pm, premaxilla; po, postorbital; pp, postparietal; prf, prefrontal; pro, prootic; prp, preparietal; pt, pterygoids; ptf, post-temporal fenestra; q, quadrate; qj, quadratojugal; rt, replacement tooth; sb, supraorbital boss; scl, sclerotic ring; smx, septomaxilla; so, supraoccipital; spl, splenial; st, stapes; sq, squamosal; sur surangular; t, tabular; v, vomer. Pictures by A. Duhamel. 3D reconstructions were made by A. Duhamel and belong to the University of the Witwatersrand. Scale bar = 1 cm.

**Figure 12:**
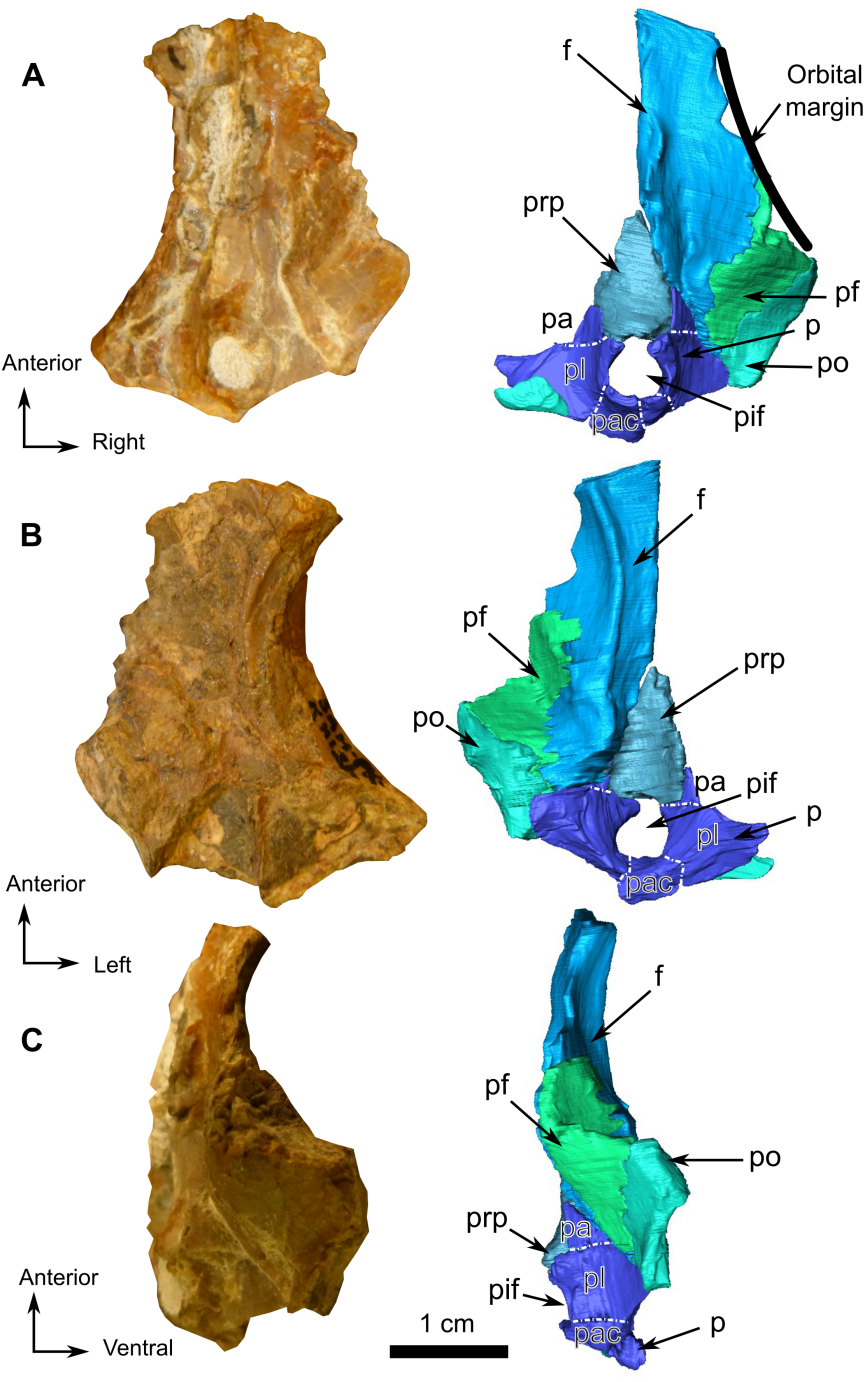
Burnetiamorpha cf. *Lophorhinus willodenensis*, skull cap, SAM-PK-K11126. Photograph (left) and 3D rendering of SAM-PK-K11126. (A) Dorsal view. (B) Ventral view. (C) Right lateral view. White dotted lines point out inner-bone sutures. Anatomical Abbreviations—ang, angular; art, articular; aso, anterior extension of the supraoccipital; bo, basioccipital; c, caniniform tooth; co, occipital condyle; cor, coronoid; d, dentary; ect, ectopterygoid; eo, exoccipital; f, frontal; fe, temporal fenestra; fm, foramen magnum; i, incisiform tooth; j, jugal; l, lacrimal; m, maxilla; n, nasal; o, orbit; op, opisthotic; p, parietal; pa, anterior portion of the parietal; pac, caudal portion of the parietal; pal, palatine; pal t, palate teeth; pao, paroccipital process; part, pre-articular; pbs, parabasisphenoid; pc, postcanine; pf, postfrontal; pif, pineal foramen; pl, lateral portion of the parietal; pm, premaxilla; po, postorbital; pp, postparietal; prf, prefrontal; pro, prootic; prp, preparietal; pt, pterygoids; ptf, post-temporal fenestra; q, quadrate; qj, quadratojugal; rt, replacement tooth; sb, supraorbital boss; scl, sclerotic ring; smx, septomaxilla; so, supraoccipital; spl, splenial; st, stapes; sq, squamosal; sur surangular; t, tabular; v, vomer. Pictures by A. Duhamel. 3D reconstructions were made by A. Duhamel and belong to the University of the Witwatersrand. Scale bar = 1 cm.

**Figure 13:**
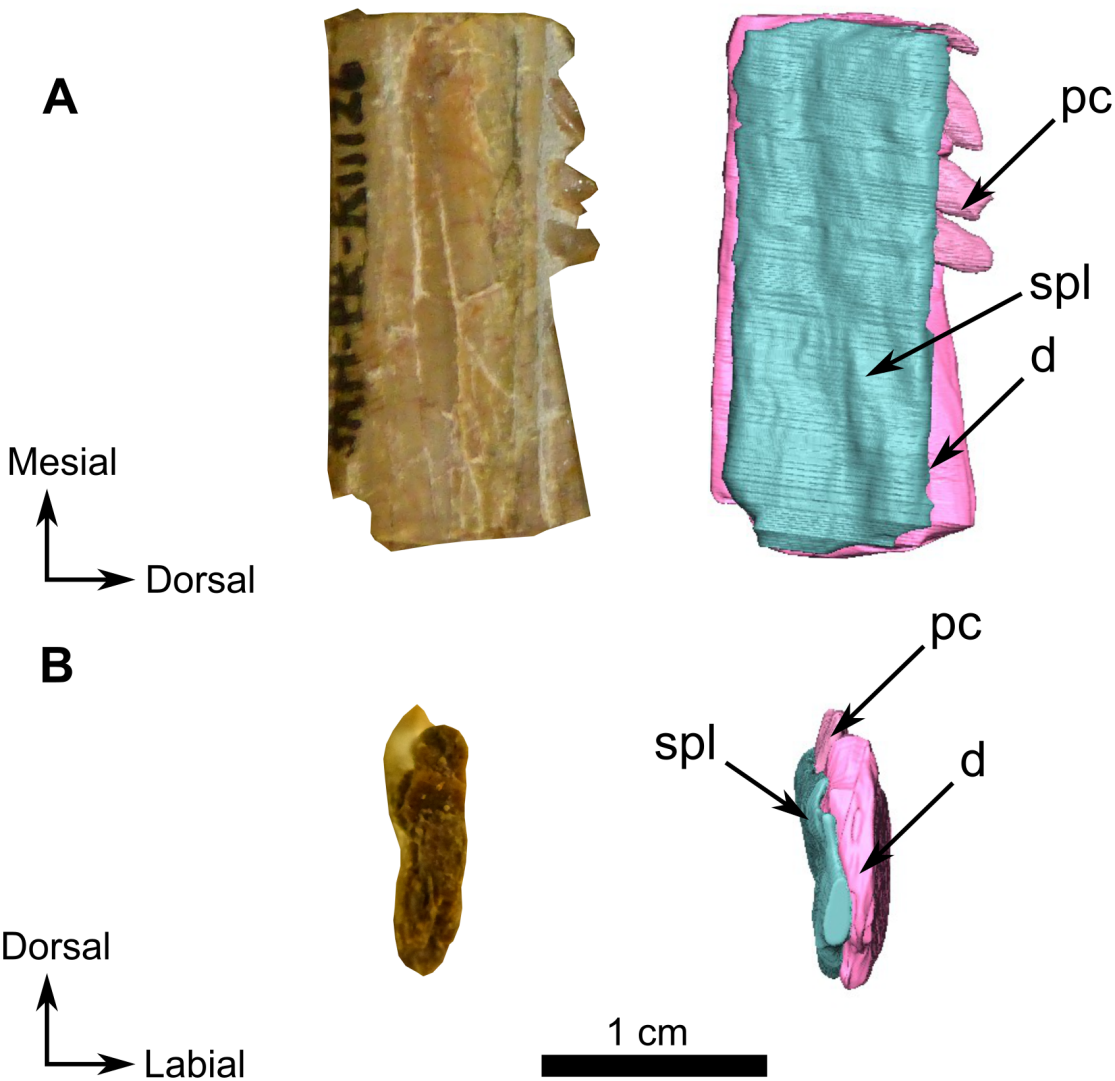
Burnetiamorpha cf. *Lophorhinus willodenensis*, right lower jaw, SAM-PK-K11126. Photograph (left) and 3D rendering of SAM-PK-K11126 (right). (A) Right lingual view. (B) Distal view. Anatomical Abbreviations—ang, angular; art, articular; aso, anterior extension of the supraoccipital; bo, basioccipital; c, caniniform tooth; co, occipital condyle; cor, coronoid; d, dentary; ect, ectopterygoid; eo, exoccipital; f, frontal; fe, temporal fenestra; fm, foramen magnum; i, incisiform tooth; j, jugal; l, lacrimal; m, maxilla; n, nasal; o, orbit; op, opisthotic; p, parietal; pa, anterior portion of the parietal; pac, caudal portion of the parietal; pal, palatine; pal t, palate teeth; pao, paroccipital process; part, pre-articular; pbs, parabasisphenoid; pc, postcanine; pf, postfrontal; pif, pineal foramen; pl, lateral portion of the parietal; pm, premaxilla; po, postorbital; pp, postparietal; prf, prefrontal; pro, prootic; prp, preparietal; pt, pterygoids; ptf, post-temporal fenestra; q, quadrate; qj, quadratojugal; rt, replacement tooth; sb, supraorbital boss; scl, sclerotic ring; smx, septomaxilla; so, supraoccipital; spl, splenial; st, stapes; sq, squamosal; sur surangular; t, tabular; v, vomer. Pictures by A. Duhamel. 3D reconstructions were made by A. Duhamel and belong to the University of the Witwatersrand. Scale bar = 1 cm.

**Description**–The description of the skull fragments of SAM-PK-K11126 is mostly based on CT data, as the delicate nature of its bones prevents further preparation. Description of the postcranial material will be the subject of a future paper. Bone sutures are readily visible making segmentation of individual bones relatively easy to accomplish (Figs. 9–13).

### Snout fragment

On the left side, most of the maxilla and the complete premaxilla are preserved as well as part of the jugal (Fig. 9A). The right side is severely damaged, and this description is thus based mostly on the left lateral side of the specimen.

The premaxilla is a short and thin bone on the anterior aspect of the snout (Fig. 9A) and accommodates six incisiform teeth. It has sutural contacts with the maxilla posterior to the last incisiform tooth, and with the vomer posteromedially through the vomerine process. In palatal view, the vomerine process of the premaxilla extends posteriorly to the level of the first caniniform tooth (Figs. 9B and 10B). Because of the presence of many crushed bones inside the snout, the morphology of the anterior part of the vomerine process of the premaxilla is uncertain (Figs. 9B and 10B). In lateral view, the premaxilla tapers posterodorsally into an elongated caudal process (Fig. 9). As the nasals are not preserved, it is not possible to evaluate the sutural contact of the premaxilla with these bones.

In lateral view the maxilla is a large bone that makes up most of the face of the isolated snout, but the bone is very thin and bears many fractures. At its anterior end, it overlaps the posterior margin of the premaxilla (Fig. 9A). Two caniniform teeth and eight postcanines are preserved on the left side (Fig. 9A). Posterodorsally the maxilla has an oblique sutural contact with the jugal which begins dorsally below the anterior margin of the orbit and continues posteroventrally to the broken zygomatic arch (Fig. 9A). In ventral view, the thin elongated maxilla has short sutural contacts with the palatine and a long contact with the ectopterygoid posteromedially (Fig. 9B).

### Palate

The anterior part of the palate is relatively well preserved on the snout, up to the posterior border of the ectopterygoid, and segmentation enabled description of the internal anatomy (Fig. 10). Because of deformation and the thin nature of some bones (particularly the dorsal lamina), some sutures were not discernible in the CT data. We present here our interpretation of the palate of SAM-PK-K11126 with some uncertainties.

The vomer is a long unpaired bone that extends almost half of the length of the palatal surface of the snout fragment (Fig. 10A). It has a longitudinal midline trough bordered by lateral ridges that join anteromedially to form a midline ridge at the level of the last incisiform, just posterior to the suture with the premaxilla (Figs. 10A and 10B). The resulting medial ridge continues anteriorly onto the premaxilla. The midline trough extends posteriorly as far as the level of the third postcanine. Posteriorly the vomer contacts the palatine and separates the palatine bosses ventrally (Fig. 10A). On its dorsal side, the vomer forms a long thin septum that separates the nasal chamber into two bilateral cavities (Figs. 10B and 10C). In lateral view, the dorsal margin of this septum slopes at a 30° angle (Fig. 10C). Posteriorly, the suture between the vomer and the palatine cannot be determined with certainty due to post-mortem damage, but it appears that the vomer thins to form a wedged contact between the two palatine bones (Fig. 10A).

The posterior half of the palatal surface comprises the paired palatine and pterygoid bones (Fig. 10A). As the right palatine bone is poorly preserved and incomplete, the description is based on the left side. The maxillary process of the palatine is triangular. It extends anteriorly along the tooth row (Fig. 10B) and tapers against the maxilla at the level of the first postcanine tooth (Fig. 9B). The palatine bosses are separated by a midline suture and a medial trough. The palatine contacts the pterygoid posteroventrally and medially (Fig. 10A). Laterally the palatine shares a long and oblique suture with the ectopterygoid. (Fig. 10A). Dorsally, a thin and high septum extends from the level of the ectopterygoid to the vomerine septum caudally (Fig. 10B, 10C and 10D). This septum may belong, at least in part, to the pterygoid, though the quality of preservation does not allow the definitive identification of a suture in CT data. Posterodorsally, the palatine overlies the pterygoid bosses (Fig. 10C).

In ventral view the ectopterygoid is a rectangular and longitudinally elongated edentulous bone located anterolateral to the lateral process of the pterygoid (Figs. 10A and 10B). The left ectopterygoid is best preserved and shows that it contacts the palatine boss medially and the maxilla laterally. Its contribution to the medial septum dorsally is uncertain (Fig. 10C). Posteromedially, the ectopterygoid borders the pterygoid boss (Fig. 10A and 10B).

In SAM-PK-K11126 only the *corpus* and quadrate ramus of the pterygoid are preserved. The quadrate ramus is visible on the occipital fragment as a thin process that contacts the quadrate posteriorly (Fig. 11D) and curves anteromedially toward the anterior margin of the parabasisphenoid (Fig. 11D). On the palatal side of the snout, the anterior part of the pterygoid *corpus* is damaged; however, it is clear that the pterygoid boss overlaps the palatine (Fig. 10C). The anterior end of the pterygoid *corpus* is separated from its counterpart by the palatine. On the left side, the pterygoid contacts the ectopterygoid laterally (Fig. 10B). The transverse process is not preserved in SAM-PK-K11126.

### Pineal region fragment

This fragment comprises the supraorbital and pineal region of the skull roof (Fig. 12). The dorsal rim of the left orbit is preserved and has no pachyostosis or supraorbital boss. The left side preserves no sutures and most of the bones could not be identified. Accordingly, the segmentation and description are mostly based on the right side (Fig. 12).

The frontal is a paired bone with a smooth dorsal surface and forms a large part of the interorbital region (Fig. 12A). A low ridge is present on the midline between the two frontals and extends posteriorly on the preparietal, and up to the pineal boss (Fig. 12A). Because the anterior portion of the frontal is not preserved, the frontal appears as a triangular bone. It tapers caudally and extends laterally to the pineal foramen as a thin caudal process contacting the parietal and preparietal medially, the postfrontal laterally, and the postorbital posteriorly (Fig. 12A). The suture between the frontal and the postfrontal appears ridged, but CT data reveal that this is due to post-mortem displacement of the frontal to artificially overlap the postfrontal (Fig. 12A). In ventral view, the frontal bears a longitudinal, medially-curved ridge extending from the level of the middle of the preparietal to the anterior part of the frontal (Fig. 12B).

The postfrontal forms the posterodorsal margin of the orbit in dorsal view (Fig. 12A) and shares a sinuous suture with the postorbital posterolaterally (Fig. 12C). In ventral view, the posterior end of the postfrontal has a short posteromedial contact with the parietal (Fig. 12B).

Only a small fragment of the postorbital is present in this skull roof fragment, posterior to the postfrontal (Fig. 12) and makes up a very short part of the posterior margin of the orbit (Fig. 12C). Anteriorly, the postorbital mostly contacts the postfrontal and also shares a short suture with the caudal process of the frontal. The postorbital has sutural contact with the parietal medially (Fig. 12B) and posterolaterally the rim of the postorbital is inset by the dorsal margin of the temporal fenestra. The squamosal is not preserved.

The preparietal is an unpaired and relatively small diamond-shaped bone on the skull roof that forms the anterior margin of the pineal foramen and tapers anteriorly between the two frontals (Fig. 12). It has a long oblique anterior sutural contact with the frontal and a short, slightly curved suture with the parietal posteriorly (Fig. 12A).

The pineal foramen is relatively large and surrounded by a prominent pineal boss formed by the preparietal anteriorly and the parietal laterally and caudally (Fig. 12A and 12B). The parietal is a paired bone that contacts the preparietal anteriorly and anterodorsally, extends onto the frontal laterally, and is overlapped by the postorbital posterolaterally (Fig. 12B). Based on CT images, it appears that the parietal comprises three parts separated by distinct sutures radiating from the pineal foramen: an anterior part, a lateral part, and a caudal part behind the pineal foramen (Fig. 12). The implications of these subdivisions are addressed in the discussion.

### Occipital fragment

The occipital part of the skull, and some of the basicranial elements are preserved on a single fragment (Fig. 11). The supraoccipital is a relatively large and unpaired bone, contributing 40% of the surface on the occiput fragment, and forming the dorsal margin of the foramen magnum (Fig. 11A). Dorsolaterally, the supraoccipital has a short contact with the remains of the postparietal (Fig. 11A). The anterior extension of the supraoccipital forms a cavity on the ventral surface to accommodate part of the brain (Fig. 11C). Ventrolaterally, the supraoccipital forms the dorsal margin of the post-temporal fenestra. Lateral and medial to the fenestra, it shares sutures with the opisthotic (Fig. 11A). Laterally the supraoccipital has a diagonal contact with the medial margin of the tabular, ventrally with the dorsal process of the basioccipital (Fig. 11A and 11B). The supraoccipital appears to surround the ovoid exoccipital (Fig. 11A) and that the supraoccipital is positioned anterior to the exoccipital. Their exact position in relation to the supraoccipital and the basioccipital cannot be accurately determined (Fig. 11A).

A broken piece of the tabular is present on the right side of the occiput in association with part of the squamosal and the quadrate (Figs. 11A and 11B). The tabular extends dorsoventrally from the top of the supraoccipital to the ventral margin of the opisthotic. It shares an anteromedial contact with the supraoccipital and contacts the opisthotic along its ventromedial margin (Fig. 11A). On its anterior side, the tabular has a slight depression to fit the anterior depressed surface of the squamosal (Fig. 11B).

Part of the dorsal process of the squamosal is positioned anterior to the tabular and the opisthotic (Figs. 11A, 11B, and 11C). The squamosal contacts the stapes posteromedially and the quadrate anteromedially.

The opisthotic is positioned on the ventrolateral side of the occiput and is shaped like a horizontal hourglass (Fig. 11A). Its dorsal concavity constitutes the ventral border of the post-temporal fenestra while its ventral concavity forms the dorsal margin of the fenestra ovalis. The opisthotic contacts the basioccipital medially, and the supraoccipital dorsomedially. Laterally, the opisthotic has a flat contact with the squamosal and is overlapped by the tabular dorsolaterally (Figs. 11A and 11B).

The basioccipital is an unpaired midline bone forming the lateral and ventral edges of the foramen magnum, and comprises the single occipital condyle (Fig. 11A). The two dorsolateral parts of the basioccipital are ovoid and in contact with the exoccipital dorsomedially, the supraoccipital medially, and the opisthotic laterally. The ventral part of the basioccipital contacts the parabasisphenoid anteriorly (Fig. 11B).

The prootic is positioned between the basioccipital and the parabasisphenoid (Figs. 11B and 11D) and shares a contact with the stapes and the supraoccipital (Fig. 11). The prootic on SAM-PK-K11126 is a tripartite bone, with a median posterior process at the level of the occipital condyle, and two lateral processes. The right lateral process is oriented toward the supraoccipital (Figs. 11B and 11D).

Only the posterior part of the quadrate is preserved on the occipital fragment (Fig. 11). Dorsally, it is in sutural contact with the opisthotic. A curved suture runs dorsolaterally along the left quadrate and separates it from the stapes (Fig. 11A). On the right quadrate, only the ventral side reaches the stapes. In right lateral view (Fig. 11B) it is evident that the quadrate underlies the squamosal and extends anterodorsally along the anterior margin of the squamosal (Fig. 11B). Anteromedially, the left quadrate contacts the quadrate ramus of the pterygoid (Figs. 11A and 11D).

The stapes is a small dumbbell-shaped bone that contacts the ventral margin of the fenestra ovalis (Fig. 11A). This description is based on the right stapes as it is the best preserved. The stapes is positioned lateral to the ventral process of the basioccipital and the prootic (Figs. 11A and 11D) and shares a sutural contact with the basioccipital and the prootic medially and the quadrate and the tabular laterally. No stapedial foramen is present.

The laterally displaced parabasisphenoid is visible on the ventral side of the fragment. Anteriorly it divides into two processes (Figs. 11B and 11D).

### Lower jaw

A small posterior portion of the left lower jaw is preserved attached to the occiput fragment (Fig. 11). No sutures could be identified but as it is articulated to the quadrate, this part of the lower jaw is probably the angular with a piece of articular (Figs. 11A and 11D). In addition to the posterior piece of left lower jaw, a small portion of the right lower jaw ramus is also preserved and bears the four distal-most postcanine teeth (Fig. 13). In dorsal view, the CT data clearly show a vertical suture extending along the three teeth sockets and the suture between the dentary and splenial (Fig. 13B).

### Dentition

**Figure 14:**
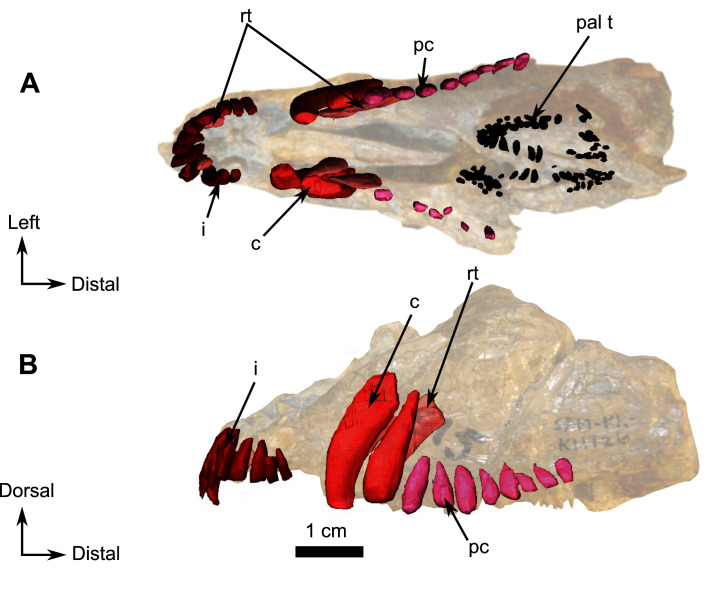
Burnetiamorpha cf. *Lophorhinus willodenensis*, upper dentition, SAM-PK-K11126. 3D rendering of the upper dentition of SAM-PK-K11126, with a photograph background of the snout. (A) Ventral view. (B) Left labial view. Anatomical Abbreviation—ang, angular; art, articular; aso, anterior extension of the supraoccipital; bo, basioccipital; c, caniniform tooth; co, occipital condyle; cor, coronoid; d, dentary; ect, ectopterygoid; eo, exoccipital; f, frontal; fe, temporal fenestra; fm, foramen magnum; i, incisiform tooth; j, jugal; l, lacrimal; m, maxilla; n, nasal; o, orbit; op, opisthotic; p, parietal; pa, anterior portion of the parietal; pac, caudal portion of the parietal; pal, palatine; pal t, palate teeth; pao, paroccipital process; part, pre-articular; pbs, parabasisphenoid; pc, postcanine; pf, postfrontal; pif, pineal foramen; pl, lateral portion of the parietal; pm, premaxilla; po, postorbital; pp, postparietal; prf, prefrontal; pro, prootic; prp, preparietal; pt, pterygoids; ptf, post-temporal fenestra; q, quadrate; qj, quadratojugal; rt, replacement tooth; sb, supraorbital boss; scl, sclerotic ring; smx, septomaxilla; so, supraoccipital; spl, splenial; st, stapes; sq, squamosal; sur surangular; t, tabular; v, vomer. Pictures by A. Duhamel. 3D reconstructions were made by A. Duhamel and belong to the University of the Witwatersrand. Scale bar = 1 cm.

On the ventral side of the snout, it is evident that six incisiform teeth are present in the premaxilla, two prominent caniniform teeth are rooted anteriorly in the maxilla, and eight postcanines are positioned caudally on the maxilla (Figs. 9 and 12A). A replacement tooth at the third incisiform position is preserved on each side (Fig. 14A). All the incisiform teeth are elongated and slender. The caniniform teeth are well exposed (Figs. 9 and 14) and have, serrations on the posterior face. The canines are curved and laterally compressed, with the distal side sharper than the mesial side. Two replacement teeth are visible medial to the caniniforms (Fig. 14). Eight postcanines are arranged in a single row on the left maxilla, while the right side shows only six poorly preserved postcanines (Figs. 9 and 14).

Posteromedially, on the body of the palatine, the prominent, anteriorly rounded, dentigerous palatine boss (Fig. 10A) bears twenty-four nubbin-like teeth arranged in a U-shaped manner along the anterior edge of the boss (Fig. 14A). On the lateral edge they are arranged in two parallel rows, but there is only a single row on the medial edge (Fig. 14A).

Nubbin-like teeth arranged in a V-shape are also present on the pterygoid boss with the apex of the V-oriented posteriorly (Fig. 10A). Fourteen teeth are visible on the CT data of the left boss, and twenty-three on the right boss.

BURNETIAMORPHA Broom, 1923

Genus *LEMUROSAURUS* Broom, 1949

*LEMUROSAURUS PRICEI* Broom, 1949

**Material**–BP/1/816 (holotype of *L. pricei*), NMQR 1702 (Sidor & Welman, 2003) and BP/1/818 (cf. *Lemurosaurus*, referred as BPI 353 in Sigogneau (1970))

**Holotype**–BP/1/816, complete skull and lower jaw.

**Figure 15:**
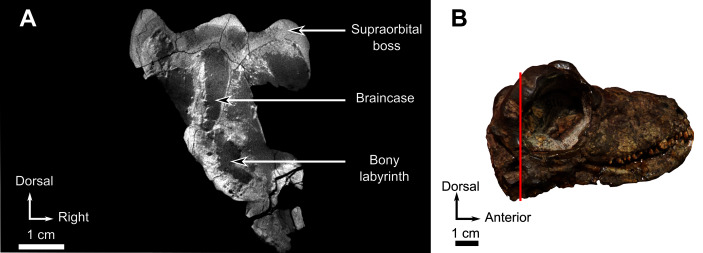
Holotype of *Lemurosaurus pricei*, skull, BP/1/816. (A) CT image at the level of the supraorbital bosses and the braincase (B). Histology of supraorbital bosses, comprising thick and dense bone, and a well-ossified braincase, indicates that the specimen is an adult. (B) Photograph of the right lateral view. Bright artefacts are caused by iron nodules. Pictures by A. Duhamel. Scan of the specimen was performed by Kudakwashe Jakata and belongs to the University of the Witwatersrand. Scale bar = 1 cm.

**Figure 16:**
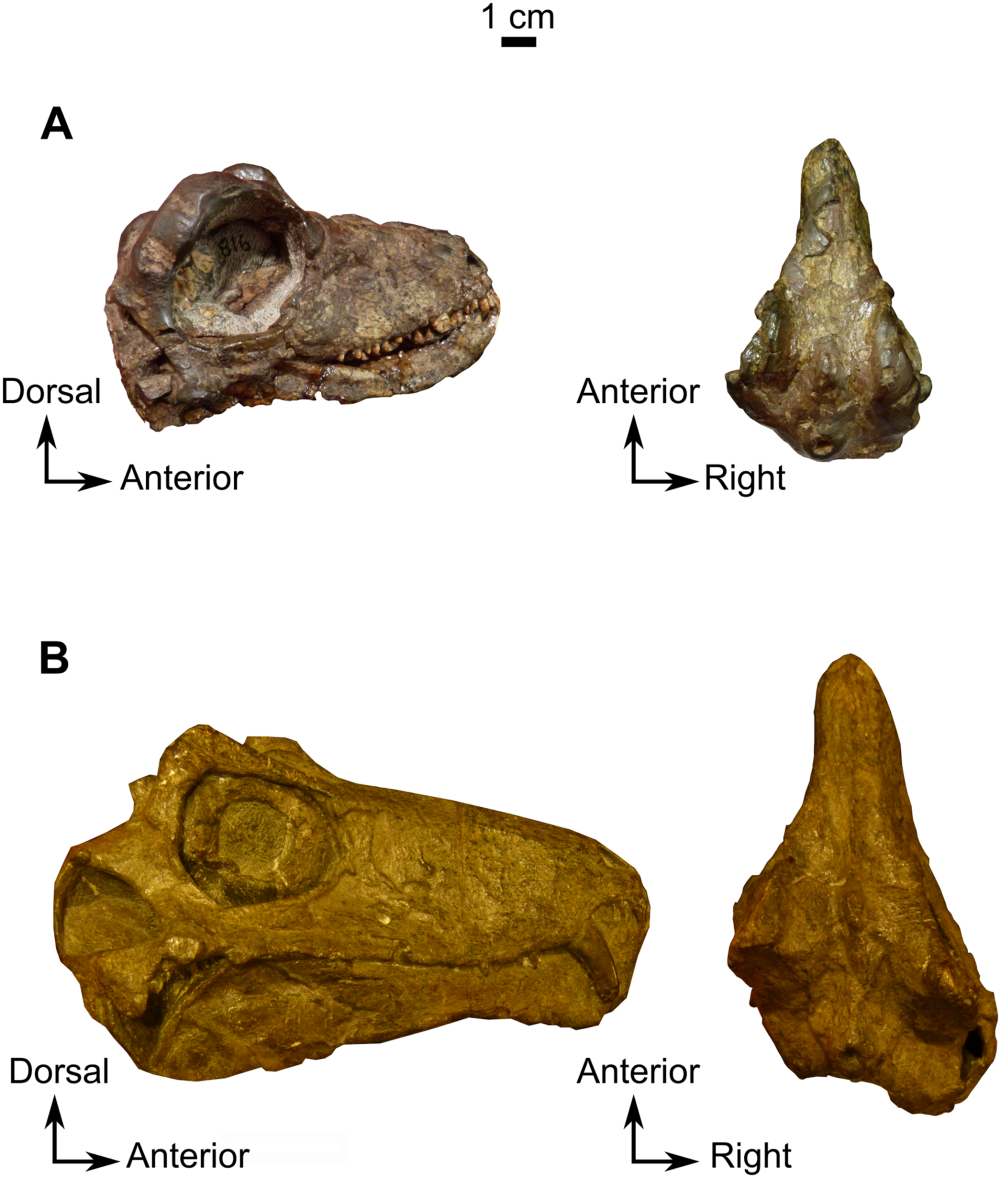
Anatomical comparison of BP/1/816 and NMQR 1702. (A) holotype of *Lemurosaurus pricei*, skull, BP/1/816, from Dorsfontein farm, Graaff-Reinet District, Eastern Cape Province, South Africa; *Cistecephalus* AZ, Beaufort Group, Karoo Supergoup. Left: right lateral view; right: dorsal view. (B) NMQR 1702, from Petersburg farm, Graaff-Reinet District, Eastern Cape Province, South Africa; *Cistecephalus* AZ, Beaufort Group, Karoo Supergroup, South Africa. Left: right lateral view; right: dorsal view. Pictures by A. Duhamel. Scale bar = 1 cm.

**Description**–Sigogneau (1970) provided a full description of BP/1/816 pointing out that the skull is small (basal skull length of 71 mm) and complete with a lower jaw. The relatively large orbit (21 mm) is suggestive of a juvenile status and it has been considered so in the literature (Sidor & Welman, 2003). The ratio of the diameter of the orbit to skull length is 0.3. This small specimen has pachyostotic and relatively large supraorbital bosses (Fig. 15). CT data (Fig. 15A) show thick and dense tissue inside the supraorbital boss instead of a thin and porous skull cap. The pineal boss is a domed structure and a round boss is present on the posterior part of the zygomatic arch. The braincase is fully formed and its walls are thick and well ossified (Benoit et al., 2017a). The two orbitosphenoids are long and fused in the midline to form a gutter that cradled the anteriormost part of the tubular brain (Benoit et al., 2017a). The orbitosphenoid sutures with the prootic caudally to close the lateral wall of the braincase. The bony labyrinth is completely separated from the brain cavity (Benoit et al., 2017a). The same degree of ossification of the braincase and bony labyrinth walls are also found in the alleged adult specimen NMQR 1702, whereas it is absent in the above-described juveniles. In CGS MJF 22, SAM-PK-K11126, and RC 55, no bony labyrinth could be segmented out because of the lack of fusion between the opisthotic and prootic and the absence of a medial wall on the inner ear capsule, and no ossified orbitosphenoid or epipterygoid are preserved. In addition, the supraorbital boss of NMQR 1702 is comparatively thinner and less developed than that of the *Lemurosaurus* holotype, despite the former being the larger specimen (Fig. 16). Sidor & Welman (2003) considered the differences between NMQR 1702 and the holotype (BP/1/816) to be the result of either intraspecific or ontogenetic variation. They gave preference to the ontogenetic explanation because of the great differences in cranial length and the comparatively larger orbit of the smaller specimen (BP/1/816), implying that BP/1/816 is a juvenile of the same species as NMQR 1702 (Sidor & Welman, 2003). From our observations and CT assisted re-assessment of these two specimens, it appears more likely that BP/1/816 and NMQR 1702 are at a similar stage of development and that BP/1/816 cannot be referred to a juvenile of the taxon represented by NMQR 1702.

## Discussion

### What are the reliable indicators of juvenility in Biarmosuchia?

Here we discuss characters that can be or have been considered in the literature as indicators of juvenile status, and their relevance in the context of biarmosuchian ontogeny at a suborder level.

### Orbit size

Specimens CGS MJF 22 and BP/1/816 have large orbits relative to their skull lengths. This character is commonly considered a juvenile characteristic in therapsids (Abdala, Flores & Giannini, 2001; Giannini et al., 2010; Jasinoski & Chinsamy-Turan, 2012; Kruger et al., 2015; Kruger, Rubidge & Abdala, 2017). In *Biarmosuchus*, the orbit remains similar in absolute size during growth from the juvenile to adult stage but reduces in size relative to skull length (Ivakhnenko, 1999, 2008). Recently, specimen BP/1/816 (*Lemurosaurus pricei*) was re-identified as a juvenile specimen because of its comparatively large orbit and overall small size (Sidor & Welman, 2003); however, as shown above, BP/1/816 displays many other traits that are indicative of a maturity. This casts doubt on the reliability of relative orbit size as an indicator of juvenility in biarmosuchians.

In extant species, a large eye diameter compared to skull length characterizes nocturnal species, as it enhances sensitivity to light in low light conditions (Hall, 2008; Heesy & Hall, 2010; Iwaniuk, Heesy & Hall, 2010; Schmitz & Wainwright, 2011). In extinct species, the dimensions of the eyeball can be inferred from those of the sclerotic ring (optical ratio) (Hall, 2008; Schmitz & Motani, 2011). The calculation of the optical ratio (a proxy to determine the light sensitivity of the eye) in synapsids enabled Angielczyk & Schmitz (2014) to determine the diel activity pattern of many species, including biarmosuchians. They concluded that most biarmosuchians had eyes adapted to either a scotopic or mesopic environment, and were thus likely not diurnal. If nocturnality was common in biarmosuchians, it is expected that many species may have developed larger orbits to accommodate larger eyeballs, and it is reasonable to hypothesize that *Lemurosaurus pricei* may be one of them. As such, a large orbit alone is not a reliable indicator of juvenility in biarmosuchians as it could be related to specific diel activity, and should be backed with other anatomical clues before concluding on a possible juvenile condition (see below).

### Tooth replacement

Specimens SAM-PK-K11126 and RC 55 have erupting caniniforms positioned lingually and posteriorly relative to the main row of teeth (Fig. 7 and 14). Until the present description replacement canines had never been observed in biarmosuchians. In dinocephalians, *Moschops, Tapinocaninus* and *Agnosaurus* show a pattern of tooth replacement (Boonstra, 1962; Whitney & Sidor, 2019; Neumann, 2020). In dicynodonts, the presence of a supernumerary tusk is considered pathological (Fröbisch, 2005; Jinnah & Rubidge, 2007; Fröbisch & Reisz, 2008). In theriodonts (Gorgonopsia, Therocephalia and Cynodontia), replacement canines are found late in ontogeny but are not found in the largest specimens (Kermack, 1956; Hopson, 1964; Van den Heever, 1980; Norton, 2020; Norton et al., 2020) except for *Thrinaxodon* (Abdala, Jasinoski & Fernandez, 2013). As such, double canines are expected to be found in, but are not exclusive to, juveniles. Specimen CGS MJF 22, and the holotypes of *Lende chiweta* and *Lemurosaurus pricei* do not show a replacement caniniform and as such, are better interpreted as mature specimens compared to SAM-PK-K11126 and RC 55 (see Norton, 2020; Norton et al., 2020).

Notably SAM-PK-K11126 has two functional upper caniniforms, which is unusual among Biarmosuchia (Sidor & Welman, 2003; Sidor & Rubidge, 2006; Day et al., 2018). In SAM-PK-K11126 (Fig. 14), it is likely that the two caniniform teeth might have been functional at the same time to some degree given that the two labial teeth are totally or partially erupted. One row of replacement teeth is positioned posterolingually to the corresponding functional caniniform (Fig. 14). The presence of two erupted maxillary caniniforms is considered a plesiomorphic condition in synapsids as sphenacodontians present continuous replacement with two functional maxillary caniniforms (Romer & Price, 1940; Van den Heever, 1980; Sigogneau-Russell, 1989; Norton, 2020; Norton et al., 2020).

### Cranial bosses and pachyostosis

**Figure 17:**
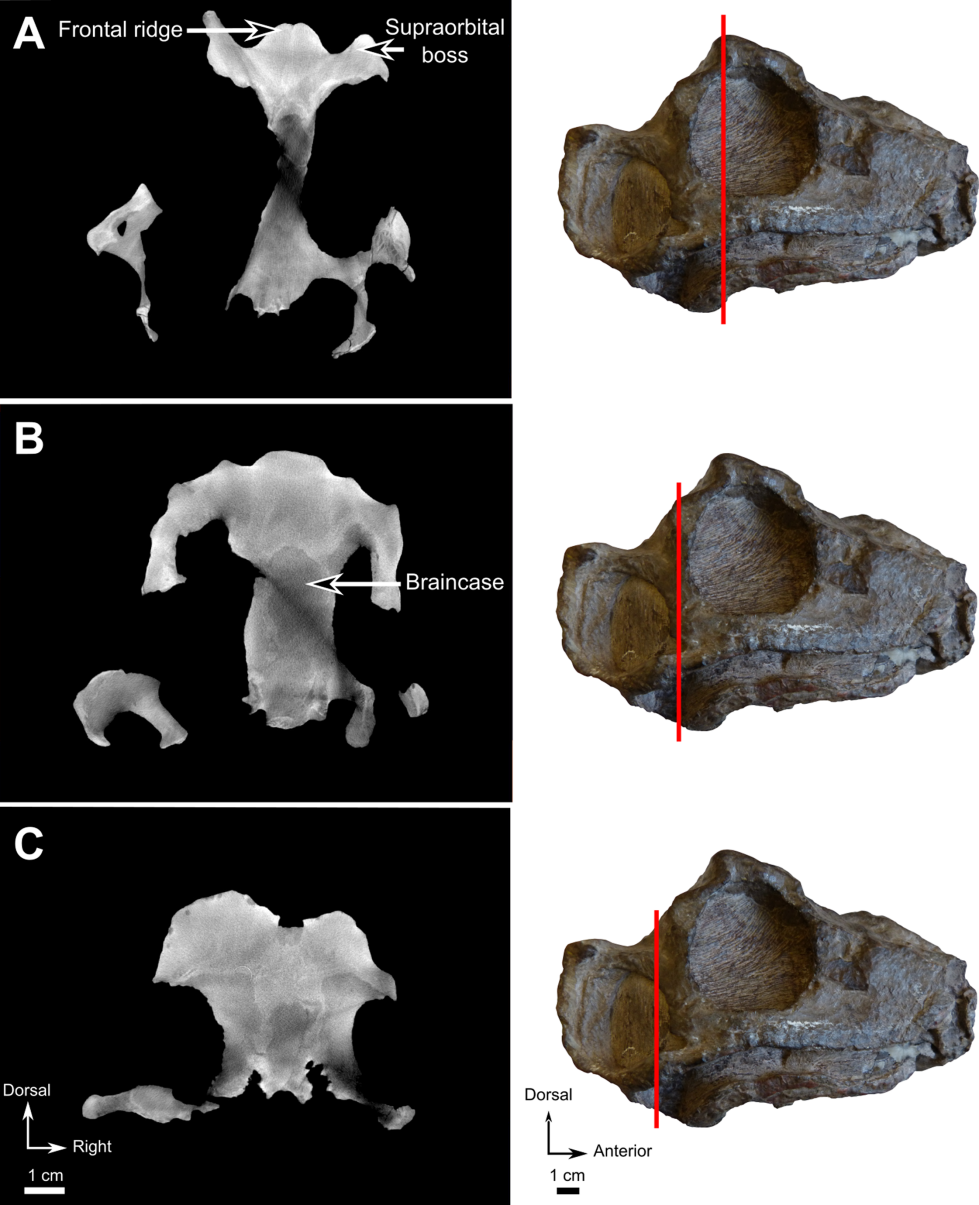
*Lende Chiweta*, skull, MAL 290. CT images (Left) and lateral view (Right); (A) at the level of the supraorbital bosses, (B) braincase and (C) foramen magnum. Bright points are artefacts resulting from the presence of iron nodules. Pictures by A. Duhamel. Scan of the specimen was performed by Kudakwashe Jakata and belongs to the University of the Witwatersrand. Scale bar = 1 cm.

Pachyostosis is a non-pathological augmentation of the volume of the bone by an increase of deposit of periosteal cortices (de Buffrénil & Rage, 1993). Among biarmosuchians, cranial pachyostosis is present in burnetiamorphs, particularly on the cranial roof (Kulik & Sidor, 2019). In CGS MJF 22, SAM-PK-K11126, and RC 55, cranial ornamentation and pachyostosis are weakly developed (Figs. 1, 8, and 12). Low development of cranial ornamentation and pachyostosis have been linked to an early ontogenetic stage in various groups of therapsids, including dinocephalians and burnetiamorphs (Estes, 1961; Van Heerden, 1972; Ivakhnenko, 2008; Horner & Goodwin, 2009; Kammerer, 2011; Liu, 2013; Cassini, Flores & Vizcaíno, 2015; Kruger, Rubidge & Abdala, 2017; Kulik & Sidor, 2019). Ivakhnenko (2008) and Kruger, Rubidge & Abdala (2017) showed that in *Titanophoneus potens* and *Anteosaurus magnificus* the fronto-nasal ridge, supraorbital bosses, and pachyostosis developed between the juvenile and the adult conditions. The formation of cranial bosses in the anteosaurian genus *Sinophoneus* was also noted as a postnatal development (Liu, 2013). A similar ontogenetic development was observed for the bosses of *Estemmenosuchus uralensis* (Ivakhnenko, 2008). In tapinocephalids, cranial pachyostosis develops while the relative size of the orbit and temporal fenestra shrinks during growth (Gregory & Broom, 1926; Boonstra & Broom, 1936; Boos et al., 2015; Neumann, 2020). Compared to the juvenile specimens studied here, adult burnetiamorphs show a higher degree of pachyostosis and cranial ornamentation (Figs. 15, 16 and 17). A low degree of pachyostosis thus appears to be linked to juvenility, whereas the higher degree of pachyostosis in MAL 290 and BP/1/816 (Figs. 15 and 17), as well as the presence of well-formed cranial bosses (Fig. 16 and 17) suggest that these specimens are a lot more mature. However, pachyostosis and cranial bosses are known to vary with phylogeny, as basal biarmosuchians display a lower degree of cranial pachyostosis and smaller cranial bosses than derived burnetiamorphs (Sidor & Rubidge, 2006; Day et al., 2018). Sexual dimorphism may also result in differences in cranial boss and pachyostosis development (Lande, 1980; Kraaijeveld, Kraaijeveld-Smit & Komdeur, 2007; Benoit et al., 2016; Gates, Organ & Zanno, 2016), although this has not been documented in biarmosuchians. As such, cranial pachyostosis should not be considered in isolation as a means to determine whether a biarmosuchian specimen is a juvenile.

### Radial vasculature pattern

In pachycephalosaur dinosaurs, the presence of radial vasculature in the bones of the pachyostotic cranial dome is indicative of juvenile status at the time of death, and overall vascularization decreases with ontogeny and the growing of pachyostosis (Schott et al., 2011). Radial vascular patterns in the cranial bones of dicynodonts have been interpreted similarly (Jasinoski & Chinsamy-Turan, 2012) and similar radially arranged vascular canals are also visible on CT images in the supraorbital bosses of CGS MJF 22 (Fig. 8B). Using thin sections and CT data, Kulik & Sidor (2019) also observed radial vasculature in the skull caps of some juvenile and sub-adult burnetiamorphs. The presence of these patterns could not be confirmed in SAM-PK-K11126 and RC 55 because their supraorbital bosses are not well preserved.

**Figure 18:**
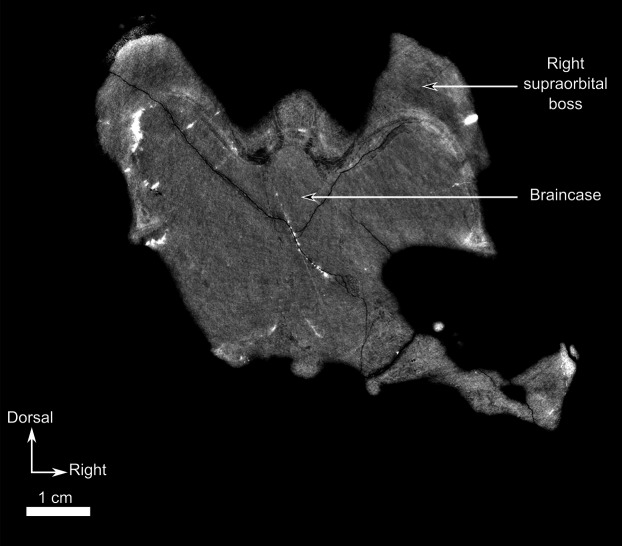
*Lemurosaurus pricei*, NMQR 1702. CT image at the level of the supraorbital bosses and the braincase. Despite the quality of the scan, the thick supraorbital bosses, the well-ossified braincase, and absence of vasculature patterns in the supraorbital bosses, indicate that the specimen is an adult. Bright artefacts are caused by iron nodules. Scan of the specimen was performed by Vincent Fernandez and belong to the European Synchrotron Radiation Facility. Scale bar = 1 cm.

A radial vascular pattern has been interpreted as an adaptation to resist tensile constraints in (Lee, 2004) but we find this an unlikely explanation in the case of CGS MJF 22 because the supraorbital boss is not an attachment point for any muscle that could have generated such tensile constraints (Benoit et al., 2016). Instead, because it occurs in combination with other characters (*e.g*., orbit size, a low degree of pachyostosis and other juvenile features), we interpret the radial vasculature patterns observed in CGS MJF 22 to be linked to the immature status of specimen. In MAL 290, NMQR 1702, and BP/1/816 (Figs. 15, 17 and 18), the supraorbital bosses are thick and dense, with no sign of a radial vasculature pattern, which suggests that their bosses were fully grown at the time of death.

### Braincase and bony labyrinth ossification

**Figure 19:**
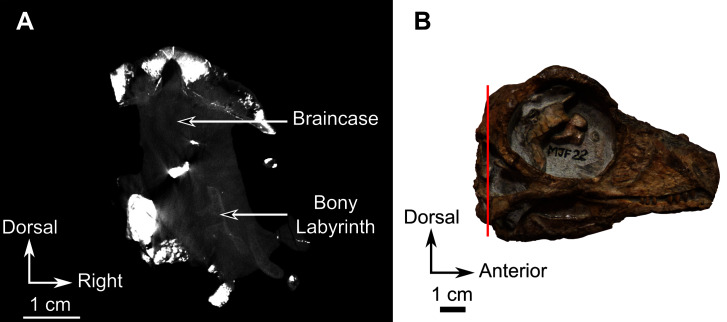
Burnetiamorpha indet., skull, CGS MJF 22. (A) CT image at the level of the braincase (B). Bony labyrinth is fused to the braincase (which itself is not ossified) which suggests incomplete ossification and juvenility. (B) Photograph of the specimen in right lateral view. Bright artefacts are caused by iron nodules. Pictures by A. Duhamel. Scan of the specimen was performed by Kudakwashe Jakata and belongs to the University of the Witwatersrand. Scale bar = 1 cm.

In CGS MJF 22, SAM-PK-K11126, and RC 55 individual cranial bones are disarticulated as most sutures are wide open, which is commonly interpreted as a juvenile trait (Estes, 1961; Kermack, 1984; Rieppel, 1992; Schaefer et al., 2009; Giere et al., 2010; Sekiya & Dong, 2010). CT images through the braincase and bony labyrinth of CGS MJF 22 (not preserved in SAM-PK-K11126 and RC 55) reveal that these anatomical structures are not well ossified as the orbitosphenoid is absent (and was likely still cartilaginous), and the prootic and opisthotic are not fused (Fig. 19). An epipterygoid was not found in any of the juvenile specimens either. This contrasts with adult burnetiamorphs in which these structures are well co-ossified and solidly sutured to each other (Fig. 15; Benoit et al., 2017b). In mammals the capsule of the bony labyrinth and the orbitosphenoid ossify early in ontogenetic development (Jeffery & Spoor, 2004; Ekdale, 2010; Koyabu et al., 2014; Spiekman & Werneburg, 2017; Sánchez-Villagra & Forasiepi, 2017), but in sauropsids this happens later and more slowly, if at all (Ngwenya et al., 2013; Neenan et al., 2019). The lack of ossification of these structures in CGS MJF 22 suggests that the development of the braincase and bony labyrinth had not yet reached maturity. The braincases of BP/1/816, MAL 290 and NMQR 1702 are fully ossified (Figs. 15, 17 and 18; Benoit et al., 2017a), which is suggestive of cranial maturity.

### Fusion of the preparietals and internal parietal sutures

**Figure 20:**
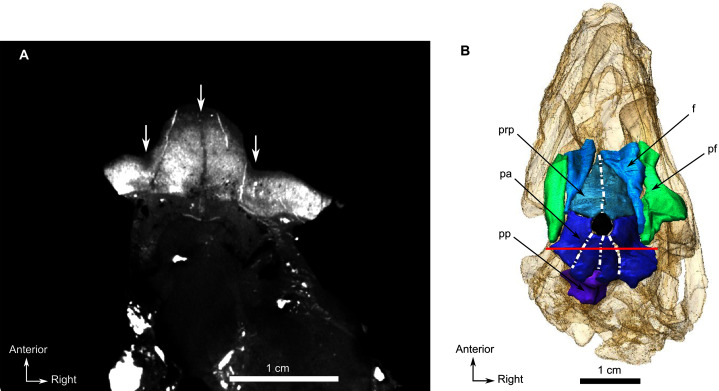
Burnetiamorpha indet., skull, CGS MJF 22. (A) CT image of a transverse section showing three symmetrical sutures, indicated by arrows, within the parietal. (B) Segmentation of the posterior part of the skull roof reveals the presence of extra-sutures in the preparietal and the parietal. The black circle represents the pineal foramen. Bright points are artefacts resulting from the presence of iron nodules. Scan of the specimen was performed by Kudakwashe Jakata and belongs to the University of the Witwatersrand. 3D reconstruction was made by A. Duhamel and belongs to the University of the Witwatersrand. Scale bar = 1 cm.

**Figure 21:**
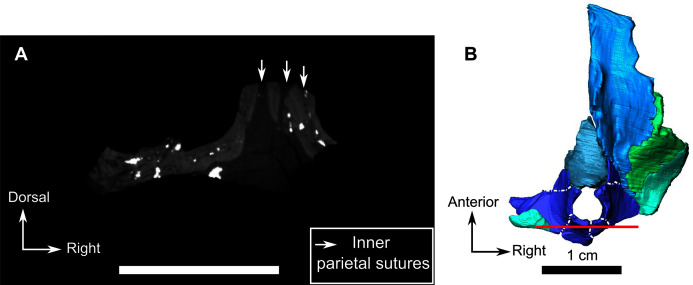
Burnetiamorph indet., dorsal piece, SAM-PK-K11126. (A) CT image of a transverse section showing three symmetrical sutures, indicated by arrows, within the parietal, at the level of the posterior border of the pineal foramen (B). (B) 3D rendering of the dorsal piece in dorsal view reveals the presence of extra-sutures in the parietal. Bright points are artefacts resulting from the presence of iron nodules. Scan of the specimen was performed by Kudakwashe Jakata and belongs to the University of the Witwatersrand. 3D reconstruction was made by A. Duhamel and belongs to the University of the Witwatersrand. Scale bar = 1 cm.

Adult biarmosuchians have an unpaired preparietal (Rubidge & Kitching, 2003; Rubidge, Sidor & Modesto, 2006; Sidor, 2003; Sidor, Hopson & Keyser, 2004; Smith, Rubidge & Sidor, 2006; Sidor & Smith, 2007; Sidor & Welman, 2003; Marilao, Kulik & Sidor, 2020). This character is present in RC 55 and SAM-PK-K11126 (Figs. 3 and 12), but not in CGS MJF 22 where the bone is paired (Figs. 8B and 20). In addition, all juvenile specimens studied here share the presence of intraparietal sutures (except, maybe RC 55, in which this condition is unclear Fig. 3A). The parietal of CGS MJF 22 is not only paired but comprises four parts (Figs. 8B and 20), while the parietal of SAM-PK-K1126 is made of six parts (Figs. 12 and 21). Based on their spatial configuration, none of these additional parts are homologous to the bones usually encountered in therapsids. As these sutures are symmetrical, they are unlikely cracks resulting from post-mortem damage (Figs. 20 and 21). From histological studies, Kulik & Sidor (2019) also observed this in the skull cap of a subadult burnetiamorph from Zambia (NHCC LB373) where the right parietal comprises two distinct bones.

Supernumerary cranial bones (called wormian bones) have been reported in adult human skulls (Bellary et al., 2013) and are formed by the addition of extra ossification centres (da Mata, da Mata & Aversi-Ferreira, 2010; Bellary et al., 2013). Veterinary studies have also reported wormian bones in dogs (Linton, 1906). However, recent studies on adult and juvenile human skulls suggest that wormian bones are more likely due to congenital pathological conditions rather than ontogenetic variation (Marti et al., 2013; Nikolova et al., 2014a). In addition, anomalous parietal sutures have been recognised in some modern human individuals (Hrdlička, 1903; Shapiro, 1972). These sutures, found on adult individuals, are perpendicular to the interparietal suture, and appear to be an anomalous rather than a juvenile feature (Shapiro, 1972). Unlike what would be expected in the case of wormian bones or post-mortem breakage, the condition in CGS MJF 22 and SAM-PK-K11126 is perfectly bilaterally symmetrical and radiates from the pineal foramen (instead of being perpendicular to the interparietal suture, or randomly distributed). As such, they are unlikely to be pathological or post-mortem damage.

Extra cranial bones have been observed in the skull of various juvenile dicynodonts and lycosuchid therocephalians (Estes, 1961; Van den Heever, 1980; Jasinoski et al., 2014; Kulik & Sidor, 2019; Marilao, Kulik & Sidor, 2020). For example, some supernumerary bones found in the nasal of a small-sized *Lystrosaurus* specimen have been tentatively homologized to the anterior process of the frontal and been hypothetically linked to their juvenile condition (Jasinoski et al., 2014). Amongst the Cynodontia, two small specimens of *Thrinaxodon liorhinus* (UCMP 42878 and UCMP 42877) possess a divided interparietal (Estes, 1961; Van Heerden, 1972). The extra intraparietal bones of CGS MJF 22 and SAM-PK-K11126 resemble the Os Incae (or Inca bones) that are present in a small fraction of modern human populations (Shapiro & Robinson, 1976; Hanihara & Ishida, 2001; Wu et al., 2011; Thanapaisal et al., 2013; Nikolova et al., 2014b). This condition usually results in a non-symmetrical division of the supraoccipital in humans; however, in some rare cases, this division can be symmetrical and results in radiating sutures, just like in CGS MJF 22 and SAM-PK-K11126 (Hanihara & Ishida, 2001: Figs. 2b and 5). In humans the presence of an interparietal is the result of the persistence of the Mendosal fontanel in adults (Wu et al., 2011), which supports the contention that the condition observed in CGS MJF 22 and SAM-PK-K11126 is a juvenile feature. The Inca bones, which in mammals normally fuse to form the interparietal bone (*e.g*. artiodactyls, hyracoids) or fuse to either the supraoccipital (*e.g*. humans) or parietal (*e.g*. possum, sirenians), comprises two to four pairs of ossification (Wu et al., 2011; Koyabu, Maier & Sánchez-Villagra, 2012; Nikolova et al., 2014b, 2014a); which is reminiscent of the condition in the parietal of CGS MJF 22.

Accordingly, we interpret the presence of intraparietal sutures and the absence of interparietal fusion as a manifestation of the juvenile status of our specimens, each resulting bone probably being an independent centre of ossification of the parietal bone normally observed in adult specimens (Figs. 20, 21). That this phenomenon is a real anatomical feature, and not the result of some taphonomic processes or CT scanning artefact, is demonstrated by the study of the histological sections made by Kulik & Sidor (2019: Fig. 1-I), who found the same results as the present work. The variable number of centres of ossifications is not abnormal given the diversity of Inca bone patterns observed in modern humans (Hanihara & Ishida, 2001; Wu et al., 2011). It appears that this condition is relatively common in juvenile burnetiamorphs (and perhaps biarmosuchians in general), but has not previously been observed in other therapsids with the the exception of the few specimens recently studied by Kulik & Sidor (2019) and Marilao, Kulik & Sidor (2020).

### Conclusion on ontogenetic characters

It is important to point out that the characters presented in this discussion cannot be considered on their own, but rather that a combination of morphological features should be considered in a specimen to assess its ontogenetic status. The general size of the specimen and its orbit diameter in comparison to the basal skull length is often a good sign of juvenility, but can also reflect the lifestyle of the animal (*e.g*. nocturnality). In modern mammals, the presence of replacement teeth is usually an indication of immaturity. By contrast, non-mammalian therapsids were likely replacing their teeth throughout their life, although it is possible that some taxa, such as *Galesaurus*, might have experienced replacement of the canine only up to the subadult stage. This variability of tooth replacement pattern between genera and species might have occurred in Biarmosuchia. The degree of development of cranial bosses and pachyostosis is traditionally interpreted as ontogenetic, but can vary between lineages and within species (*e.g*. sexual dimorphism). The degree of ossification of the braincase region is rather a strong argument to assess the ontogenetic stage of the specimen, but like in extant species, the timing of ossification of the different internal bones is taxon dependant. The presence of intra-bone sutures is rarely observed in extinct and extant species, especially on the parietal, but, when not pathological, this has been observed in immature specimens only.

### A hypothetical biarmosuchian ontogenetic series

As demonstrated above there are a number of clues from cranial morphology that, when considered together, enable recognition of a juvenile burnetiamorph: the presence of two caniniform teeth on the upper jaw; incomplete cranial fusion; low degree of pachyostosis and cranial ornamentation (bosses and ridges small or absent); and the presence of a paired preparietal and subdivisions of the parietal. A sizable orbit relative to skull length and a relatively short snout are not reliable juvenile characters on their own as they can be the result of interspecific allometry.

**Figure 22:**
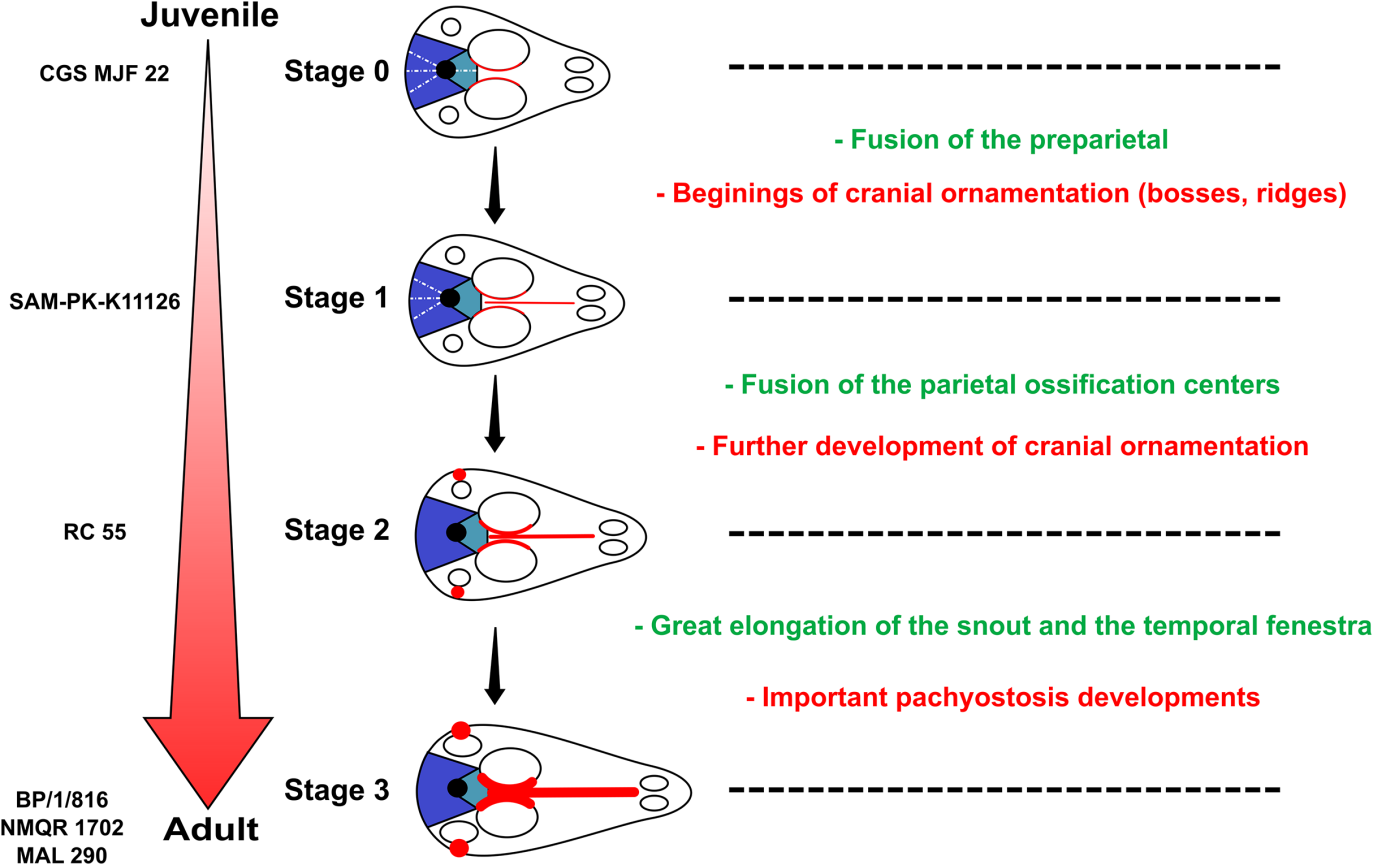
A hypothetical biarmosuchian ontogenetic series. Characters present in only Burnetiamorpha are written and drawn in red. Characters present in all biarmosuchian taxa are written in green. Figure by A. Duhamel.

Here we propose a hypothetical ontogenetic sequence of the Biarmosuchia based on the anatomical data from the actual specimens studied and literature presented above (Fig. 22). It is challenging to build a complete ontogenetic series of an extinct higher ranked taxon as the order of closure of the sutures is likely to be variable among the lower ranked taxa. As such, our hypothetical model focuses on the occurrence of different centres of ossification, the presence and relative size of cranial bosses and ridges, and the overall proportions of the different parts of the skull. Two ontogenetic directions are reconstructed depending on whether the species considered is a burnetiamorph or a non-burnetiamorph biarmosuchian (Fig. 22). In green are reconstructed stages that we postulate for all biarmosuchians and in red are ontogenetic stages that would pertain only to burnetiamorphs.

Beginning with a hypothetical juvenile stage (stage 0 on Fig. 22; sutures visible within the parietal, preparietal paired, no cranial ornamentation visible, replacement caniniforms present as in CGS MJF 22), we identified two different juvenile stages before the adult stage. In stage 1 (Fig. 22), the preparietal fuses, and the supraorbital bosses and naso-frontal ridge then begins to grow (only in burnetiamorphs). This stage is exemplified by SAM-PK-K11126. In stage 2 (Fig. 22), the intraparietal sutures may fuse, and cranial ornamentation becomes larger (in burnetiamorphs only). According to the model, this stage corresponds to the status of RC 55. Finally, the possible adult stage (stage 3 in Fig. 22) is marked by the absence of replacement caniniforms, significant elongation of the snout and enlargement of the temporal fenestra. In burnetiamorphs cranial ornaments lose their radial vasculature, the skull roof is fully pachyostotic, and the braincase and bony labyrinth become completely ossified. According to this model, the development of cranial pachyostosis occurs after complete ossification of the preparietal and parietal, which concurs with the conclusions made by Kulik & Sidor (2019).

This hypothetical series could be tested in the future by the discovery of a single species ontogenetic series.

### Reconsideration of *Lemurosaurus* Broom, 1949?

In contrast to the situation in the juvenile specimens described above, both the braincase and the bony labyrinth of BP/1/816 (holotype of *Lemurosaurus pricei*) are well ossified (Fig. 15; Benoit et al., 2017a) and they are even more extensively ossified than in the largest specimen (NMQR 1702) attributed to *Lemurosaurus pricei* (Fig. 19; Benoit et al., 2017b). Most cranial sutures of the holotype are completely fused and cannot be determined, even on the CT images (Fig. 15). In contrast to the juvenile biarmosuchian specimens described above, the supraorbital bosses are well developed, show no radial vasculature (Figs. 15 and 16A). More importantly, no replacement canine is present, which is only found otherwise in the most mature (such as the holotype of *Lende chiweta*), and largest specimens in other therapsid taxa (Norton, 2020). This supports the idea that the holotype of *Lemurosaurus pricei* (BP/1/816), despite having very large orbits and a relatively short snout, is an adult (contra Sidor & Welman, 2003). As stated above, the large size of the orbit is not a reliable indicator of juvenility in biarmosuchians. The only characters left supporting that BP/1/816 may be a juvenile are thus its small cranial length and short snout, two characters that are also expected to occur in dwarf species of a given taxon (Reynoso & Clark, 1998). As such, given the comparatively large amount of evidence suggesting that BP/1/816 is an adult, the juvenile hypothesis is untenable. Specimen NMQR 1702, one of the best preserved biarmosuchian specimens, comprises a large skull with lower jaw that was described as an adult specimen of *Lemurosaurus pricei* by Sidor & Welman (2003). Compared to the holotype of *Lemurosaurus* (BP/1/816), NMQR 1702 has a much longer snout (115 mm) and the size of the orbit is smaller (35 mm) relative to skull length, with an orbit to basal skull length ratio of 0.3 (Fig. 16). In contrast to BP/1/816, which has six postcanines, the maxilla of NMQR 1702 bears only three or four teeth (Sidor & Welman, 2003). If BP/1/816 is a juvenile representative of the same taxon as NMQR 1702, this would imply that the number of post-canine teeth decreasd during ontogeny, whereas jaw length increased. Though counterintuitive, such a reduction has been anecdotally hypothesized in gorgonopsians (Sigogneau (1970), who, in contrast, found that the dental formula remains constant) but dedicated studies are lacking. Two zygomatic bosses are present in NMQR1702, whereas there is only one in BP/1/816 (Fig. 16). The supraorbital boss of NMQR 1702 is comparatively thinner and less developed than that of the *Lemurosaurus* holotype (Fig. 16). In the holotype the highest point on the skull roof is above the centre of the orbit, whereas the highest point in NMQR 1702 is behind the orbit (Fig. 16). The skull roof of NMQR 1702 has a long midline crest extending from the nasal to the parietal foramen, whereas the holotype of *Lemurosaurus pricei* has only a little boss at the level of the orbit (Fig. 16). The pineal boss of NMQR 1702 is a well-defined chimney, whereas that of the *Lemurosaurus* holotype is a smooth dome-like structure (Fig. 16).

In occipital view, the external occipital ridge of NMQR 1702 extends ventrally across the postparietal and supraoccipital from the skull roof to the foramen magnum. In the holotype, this ridge is confined to the postparietal. In addition, NMQR 1702 possesses a nubbin-like boss on the dorsal apex of the lateral temporal fenestra; a low-developed boss on the anteroventral margin of the temporal fenestra, and two bosses on the zygomatic bar; characters that are absent on the holotype.

Considering the number and nature of the anatomical differences between BP/1/816 and NMQR 1702, and given that BP/1/816 might be an adult individual, it is unlikely that these differences represent intraspecific variation and it is more likely that NMQR 1702 represents a different taxon, distinct from *Lemurosaurus pricei*. The creation of a new nomenclatorial combination for NMQR 1702 might be taken into consideration in the future.

Similarly, MAL 290, the holotype of *Lende Chiweta*, which has relatively large orbits in comparison to the skull length, was previously identified as a possible juvenile (Kruger et al., 2015). However, it displays well-developed cranial ornamentation and no visible replacement caniniforms, which are probably indicative of an adult, very mature age (Fig. 17). Along with BP1/816, MAL 290 suggests that a paedomorphic cranial morphology (large orbit, short snout) might have been common among adult burnetiamorphs. As discussed above, large orbits may have been an adaptation to a nocturnal lifestyle in biarmosuchians (Angielczyk & Schmitz, 2014), but further study will be necessary to better understand the cause of such dramatic interspecific allometry.

## Conclusion

The discovery of new immature biarmosuchian cranial material and its study has lead to a better understanding of ontogenetic variation within the clade. We found that the presence of multiple centres of ossification in the bones of the skull roof is unique to juvenile biarmosuchians and a reliable indicator of an immature individual in addition to the presence of a replacement canine, poorly developed cranial ornamentation and pachyostosis, and poorly ossified braincase and bony labyrinth. In contrast, the reliability of other previously recognised possible juvenile characters, such as the presence of relatively large orbits, is questioned. Some of these juvenile features are currently used in character matrices for phylogenetic analysis and need to be reconsidered in light of the ontogenetic and intraspecific variations highlighted in this contribution.

## Institutional abbreviations

AMNH: American Museum of Natural History, New York, USA
BP: Evolutionary Studies Institute (formerly the Bernard Price Institute for Paleontological Research), University of the Witwatersrand, Johannesburg, South Africa
CGS: Council for Geosciences, Pretoria, South Africa
MAL: Malawi Department of Antiquities Collection, Lilongwe and Nguludi, Malawi
NHCC: National Heritage Conservation Commission, Lusaka, Zambia
NMQR: National Museum, Bloemfontein, South Africa
PIN: Paleontological Institute, Moscow, Russia
RC: Rubidge Collection, Graaff-Reinet, South Africa
SAM: Iziko South African Museum, Cape Town, South Africa

**Anatomical abbreviation**
ang: angular
art: articular
aso: anterior extension of the supraoccipital
bo: basioccipital
c: caniniform tooth
co: occipital condyle
cor: coronoid
d: dentary
ect: ectopterygoid
eo: exoccipital
f: frontal
fe: temporal fenestra
fm: foramen magnum
i: incisiform tooth
j: jugal
l: lacrimal
m: maxilla
n: nasal
o: orbit
op: opisthotic
p: parietal
pa: anterior portion of the parietal
pac: caudal portion of the parietal
pal: palatine
pal t: palatal teeth
pao: paroccipital process
part: pre-articular
pbs: parabasisphenoid
pc: postcanine
pf: postfrontal
pif: pineal foramen
pl: lateral portion of the parietal
pm: premaxilla
po: postorbital
pp: postparietal
prf: prefrontal
pro: prootic
prp: preparietal
pt: pterygoids
ptf: post-temporal fenestra
q: quadrate
qj: quadratojugal
rt: replacement tooth
sb: supraorbital boss
scl: sclerotic ring
smx: septomaxilla
so: supraoccipital
spl: splenial
st: stapes
sq: squamosal
sur: surangular
t: tabular
v: vomer

## Supplemental Information

10.7717/peerj.11866/supp-1 Supplemental Information 1 Surface files of the newly described specimens.

10.7717/peerj.11866/supp-2 Supplemental Information 2 Burnetiamorpha indet., skull, CGP MJF 22. (**A**) CT-images showing parietal sutures and centres of ossification, (**B**) at the corresponding parietal transversal section. Occipital view. Scale bar = 1 cm.

10.7717/peerj.11866/supp-3 Supplemental Information 3 Burnetiamorpha indet., skull, CGP MJF 22. (**A**) CT-images showing parietal sutures and centres of ossification, (**B**) at the corresponding three parietal longitudinal sections. Dorsal view. Scale bar = 1 cm.

10.7717/peerj.11866/supp-4 Supplemental Information 4 Holotype of *Rubidgina angusticeps*, here considered Biarmosuchia indet., RC 55. (**A**) CT-images at the level of the pineal foramen and the braincase, (**B**) showing the general ossification of the braincase of the specimen. Scale bar=5mm.

10.7717/peerj.11866/supp-5 Supplemental Information 5 Burnetiamorpha cf. *Lophorhinus willodenensis*, SAM-PK-K11126. (**A**) CT-image showing the general braincase ossification of the specimen, (**B**) at the level of the basioccipital. Scale bar = 1 cm.

## Additional Information and Declarations

### Data Availability

The following information was supplied regarding data availability: The 3D data, surface files from the 3D reconstruction, in .pty, arranged by specimen number, are available in the Supplemental Files. The data are also available at MorphoBank: DOI 10.7934/P4003. The raw CT data are stored at the Evolutionary Studies Institute and are available upon request to the authors by email alienor.duhamel@ens-lyon.org. The specimens are in the following locations: - CGS MJF 22 (collection and access number) is stored at the Council for Geosciences in Pretoria, South Africa. - RC 55 (collection and access number) is stored in the Rubidge Collection, Graaff-Reinet, South Africa. - SAM-PK-K11126 (collection and access number) is stored at Iziko South African Museum, Cape Town, South Africa. - BP/1/816 (collection and access number) is stored at the Evolutionary Studies Institute, Johannesburg, South Africa. - NMQR 1702 (collection and access number) is stored at the National Museum, Bloemfontein, South Africa. - MAL 290 (collection and access number) is stored at the Malawi Department of Antiquities Collection, Lilongwe, Malawi.
