# Supplementary figures and images for: Computed Tomography elucidates ontogeny within the basal therapsid clade Biarmosuchia
Source: PeerJ. 2021 Aug 26;9:e11866. doi: 10.7717/peerj.11866 (PMC8403480; doi:10.7717/peerj.11866)

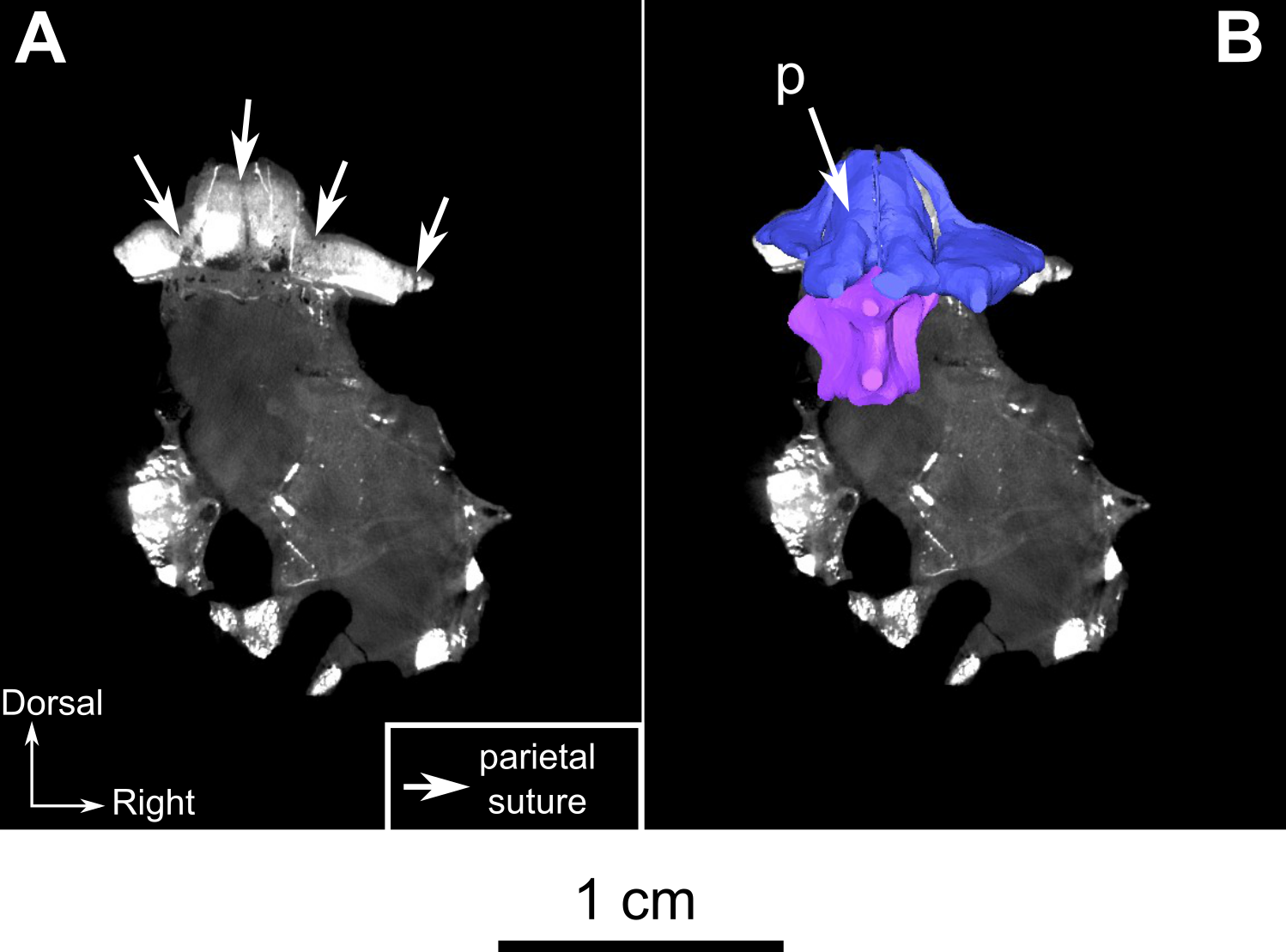

Supplement: Supplemental Information 2 — (A) CT-images showing parietal sutures and centres of ossification, (B) at the corresponding parietal transversal section. Occipital view. Scale bar = 1 cm. [file peerj-09-11866-s002.png]

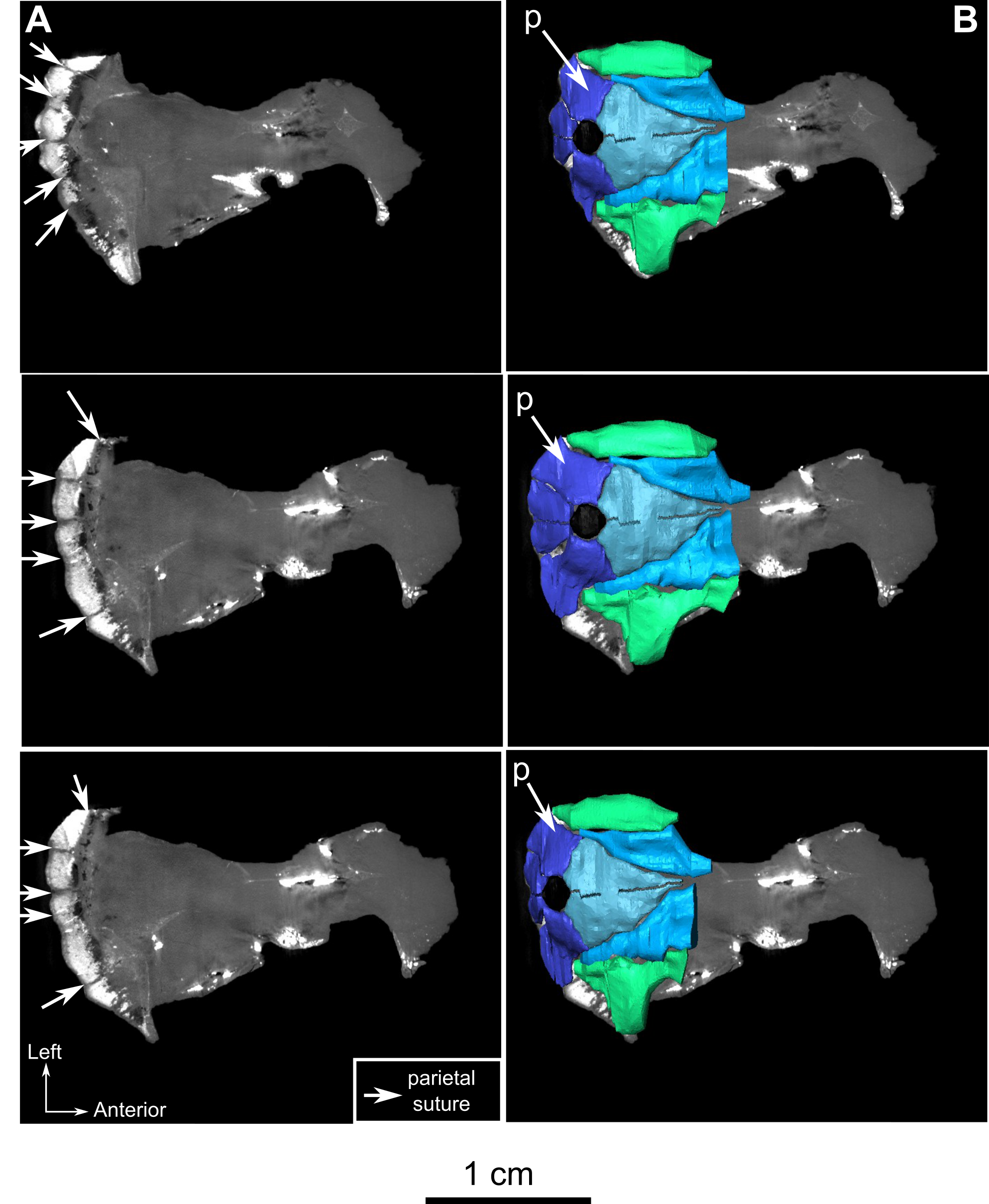

Supplement: Supplemental Information 3 — (A) CT-images showing parietal sutures and centres of ossification, (B) at the corresponding three parietal longitudinal sections. Dorsal view. Scale bar = 1 cm. [file peerj-09-11866-s003.png]

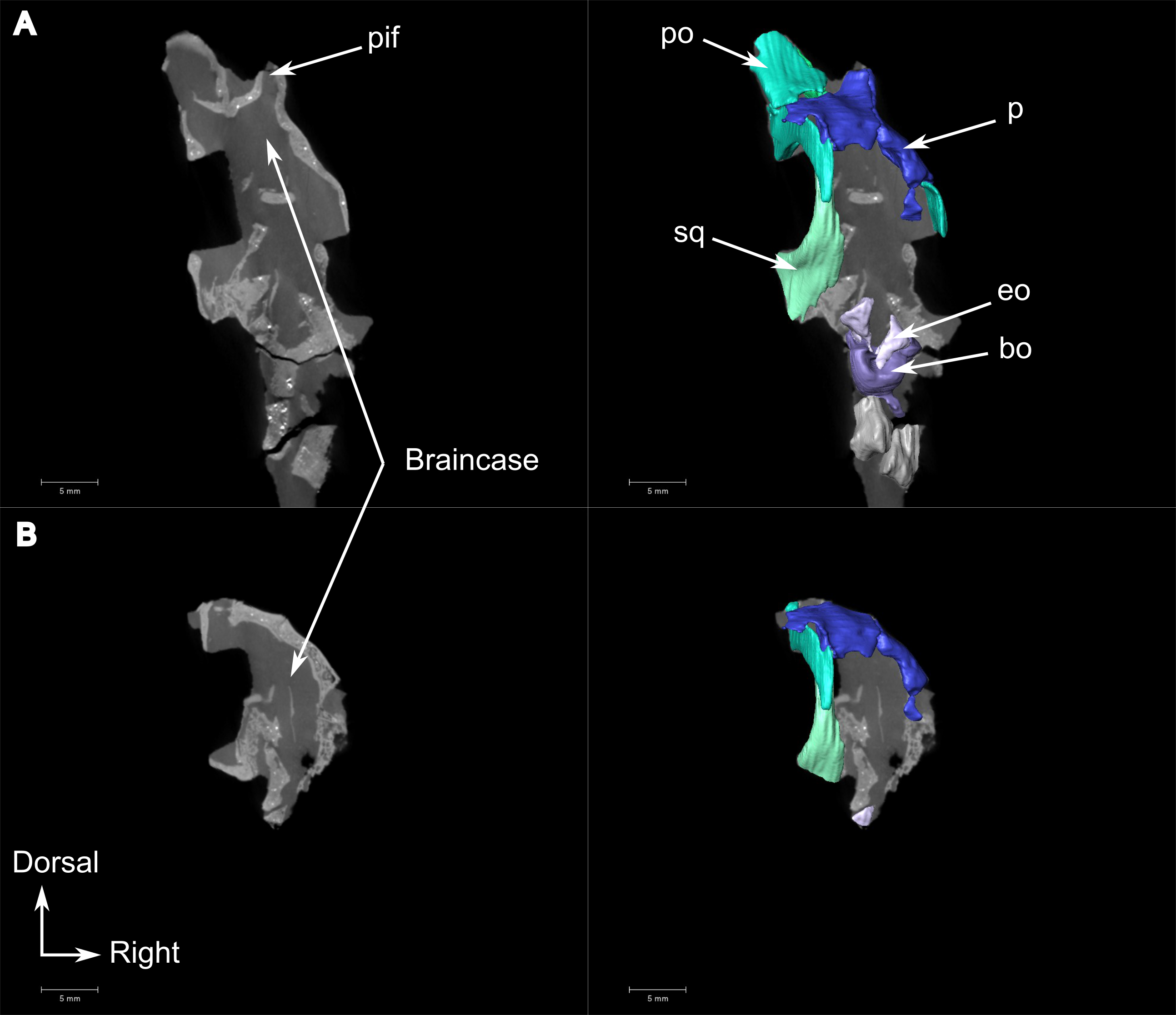

Supplement: Supplemental Information 4 — (A) CT-images at the level of the pineal foramen and the braincase, (B) showing the general ossification of the braincase of the specimen. Scale bar=5mm. [file peerj-09-11866-s004.png]

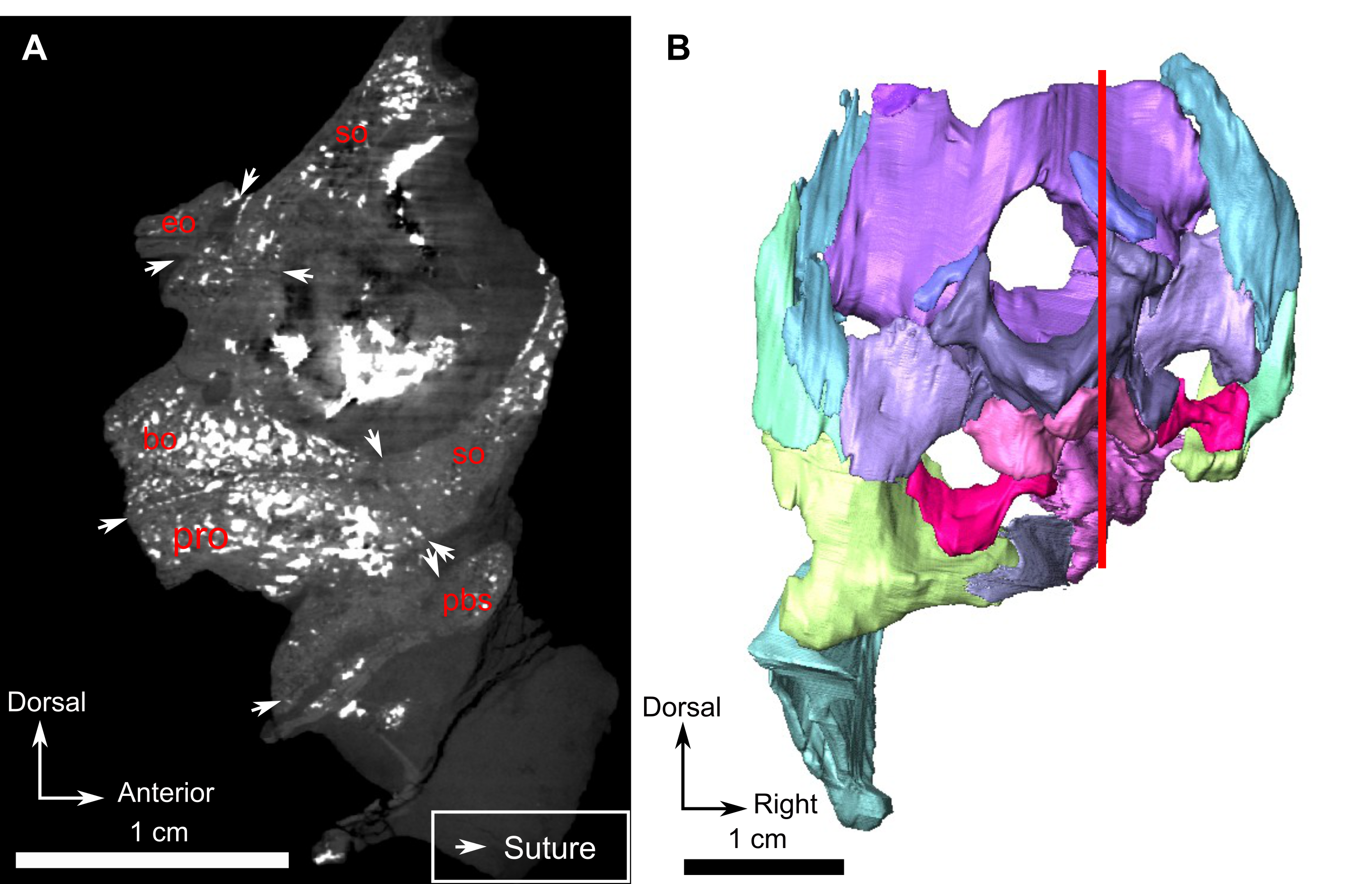

Supplement: Supplemental Information 5 — (A) CT-image showing the general braincase ossification of the specimen, (B) at the level of the basioccipital. Scale bar = 1 cm. [file peerj-09-11866-s005.png]
